# Microbiota-Dependent Alleviation of Ulcerative Colitis by Liubao Tea: Integrated Insights into SCFA and Arachidonic Acid Metabolism

**DOI:** 10.3390/foods15173085

**Published:** 2026-08-31

**Authors:** Xiao Yang, Song Xu, Ying Tong, Jichu Luo, Xixing Fang, Jiaxing Du, Changyuan Zhou, Guangnian Hu, Bao Yang, Qisong Zhang

**Affiliations:** 1 Hubei Provincial Key Laboratory of Occurrence and Intervention of Rheumatic Diseases, Hubei Minzu University, Enshi 445000, China; yangx5679@163.com (X.Y.); fangxixing2020@163.com (X.F.); dujiaxing1126@163.com (J.D.); zhouchangyuan@hbmzu.edu.cn (C.Z.); h997445219@163.com (G.H.); 2 Medical School, Hubei Minzu University, Enshi 445000, China; 3 Guangxi Key Laboratory of Special Biomedicine, School of Medicine, Guangxi University, Nanning 530004, China; fighting68_xusong@163.com (S.X.); tongying0206@163.com (Y.T.); luojichu1999@163.com (J.L.)

**Keywords:** Liubao tea, ulcerative colitis, gut microbiota, short-chain fatty acid, arachidonic acid

## Abstract

(1) Background: Ulcerative colitis (UC) is a chronic inflammatory bowel disease associated with gut microbiota dysbiosis and metabolic perturbations. Although Liubao tea (LBT) has gastroprotective benefits, the precise mechanisms by which LBT extract (LBTE) alleviates UC by orchestrating microbial and metabolic homeostasis remain poorly understood. (2) Methods: A DSS-induced UC mouse model was used to evaluate LBTE efficacy. Serum pharmacochemistry, untargeted metabolomics, 16S rRNA sequencing, and targeted SCFA metabolomics were integrated to characterize absorbable active constituents, metabolic shifts, and gut microbiota landscapes. SCFA- and arachidonic acid metabolism-related targets were validated by RT-qPCR and Western blotting. PGF models and FMT were used to assess the causal role of gut microbiota in LBTE-mediated efficacy. (3) Results: LBTE preserved colon length and mucosal integrity while reducing IL-6, TNF-α, IL-1β, and oxidative stress. It enriched SCFA-producing genera and increased colonic butyric and valeric acids, activating GPR41/GPR109A signaling, upregulating ZO-1 and occludin, and strengthening the intestinal barrier. LBTE also downregulated PTGS2 and ALOX5, restored PTGS1 and CYP3A11, and inhibited NF-κB signaling. These effects were weakened in PGF mice but reproduced by FMT from LBTE-treated donors, confirming microbiota-dependent protection. (4) Conclusions: LBTE may serve as a complementary strategy for UC prevention and management.

## 1. Introduction

Ulcerative colitis (UC), a primary clinical manifestation of inflammatory bowel disease (IBD), has emerged as a significant global public health challenge with an escalating worldwide incidence [1,2]. Characterized by chronic intestinal inflammation, compromised epithelial barrier integrity, and profound gut microbiota dysbiosis, UC severely impairs the quality of life and imposes a substantial socioeconomic burden. Owing to its multifaceted pathogenesis and the lack of a definitive cure, UC requires long-term pharmacological management to control clinical symptoms [3]. Current therapeutic strategies, including aminosalicylates (e.g., 5-aminosalicylic acid, sulfasalazine), immunosuppressants (e.g., azathioprine, cyclosporine), and monoclonal antibody-based biologics (e.g., infliximab, vedolizumab, ustekinumab), along with bionanomaterials employed as therapeutic delivery vehicles, primarily target symptom relief and inflammatory suppression [4,5,6]. However, their clinical utility is frequently limited by systemic toxicities, high non-response rates, and transient remissions. Consequently, there is an urgent demand for novel therapeutic strategies for the effective treatment of UC. Gut microbiota dysbiosis is widely acknowledged to play a critical role in the pathogenesis and progression of UC. Accordingly, reshaping gut microecological homeostasis and restoring microbial metabolic functions have emerged as a pivotal research direction for the development of novel therapeutic strategies against UC [7]. Studies have shown that herbal products, such as *Osbeckia opipara* extract, mulberry anthocyanins, *Phyllanthus emblica* fruit polysaccharides, and *Codonopsis pilosula* polysaccharide, can promote the proliferation of beneficial gut bacteria, and some of them alleviate UC via this mechanism [8,9,10,11]. Collectively, these findings highlight the therapeutic promise of herbal products in UC management, positioning them as promising candidates for microbiome-targeted therapies through their dual ability to suppress inflammation and restore gut homeostasis [12].

Liubao tea (LBT), a traditional post-fermented dark tea derived from *Camellia sinensis* (L.) Kuntze, is rich in bioactive constituents, including polyphenols, pheophytins, alkaloids, and polysaccharides, which contribute to its antioxidant and anti-inflammatory activities [13,14]. LBT has also been reported to modulate gut microbiota composition and alleviate intestinal pathological alterations [15]. Our previous studies demonstrated that Liubao tea extract (LBTE) reshaped the gut microbial landscape in experimental models of hyperlipidemia and non-alcoholic fatty liver disease [16,17]. While these findings do not directly confirm the therapeutic efficacy of LBTE in UC, they underscore its broad capacity to restore gut microbial and metabolic homeostasis across different pathological conditions. Supporting this line of evidence, recent investigations have reported that aqueous LBT effectively ameliorates UC-related pathology in a rat model [18]. Nonetheless, the precise mechanisms, particularly the microbiota-dependent metabolic interactions between host and microbes, remain to be fully elucidated.

The gut microbiota-derived metabolites, particularly short-chain fatty acids (SCFAs) such as acetate, propionate, and butyrate, are pivotal regulators of colonic homeostasis and systemic inflammatory responses. UC patients typically exhibit a depletion of SCFA-producing bacteria, which contributes to impaired mucosal barrier function and persistent intestinal inflammation [19]. Beyond serving as energy substrates for colonocytes, SCFAs function as signaling molecules that exert anti-inflammatory and immunoregulatory effects through G-protein-coupled receptors, including GPR41 and GPR109A, and through inhibition of histone deacetylases [20]. These effects may further influence cytokine and lipid-mediator responses, providing a potential biological link between microbiota-derived SCFAs and host inflammatory lipid metabolism. In addition, arachidonic acid metabolism represents another critical metabolic axis in UC. Arachidonic acid is metabolized through cyclooxygenase (COX), lipoxygenase (LOX), and cytochrome P450 (CYP450) to generate bioactive eicosanoids, including prostaglandins, hydroxyeicosatetraenoic acids, and leukotrienes, which contribute to intestinal inflammatory responses [21]. Notably, SCFA-mediated anti-inflammatory signaling and arachidonic acid-derived lipid mediators may converge on common inflammatory pathways, including NF-κB and MAPK signaling [22]. Therefore, gut microbiota dysbiosis may simultaneously reduce protective SCFA signaling and favor a pro-inflammatory host lipid-metabolic environment. From this perspective, SCFA and arachidonic acid metabolism represent interconnected microbial and host metabolic dimensions of UC pathogenesis, providing a biological rationale for investigating their coordinated modulation by LBTE. However, whether LBTE alleviates UC through the concurrent regulation of these two metabolic axes remains unclear.

This study systematically evaluated the therapeutic potential of LBTE in a dextran sodium sulfate (DSS)-induced UC mouse model, focusing on microbiota-regulated SCFA and host arachidonic acid metabolism. An integrative strategy coupling serum pharmacochemistry and network pharmacology was employed to identify the primary bioactive constituents and their potential core targets governing UC pathogenesis. By utilizing a highly orchestrated multi-omics network that intertwines 16S rRNA sequencing, targeted SCFA profiling, untargeted metabolomics, alongside stringent molecular validation via RT-qPCR and Western blotting, the present study comprehensively characterized the multi-dimensional therapeutic effects of LBTE against UC. Furthermore, pseudo-germ-free (PGF) mouse models and fecal microbiota transplantation (FMT) were employed to solidify the causal necessity of the gut microbiota and its derived metabotypes in driving LBTE-mediated colonic protection. Building on a previous study that established the therapeutic efficacy of LBT extract against UC symptoms, the present study advances the field by demonstrating microbiota dependence, and by integrating microbial SCFAs metabolism with host arachidonic acid metabolism, thereby providing a mechanistic framework for LBTE-mediated protection against UC.

## 2. Materials and Methods

### 2.1. Materials and Reagents

The LBT was provided by China Tea Co., Ltd. (Wuzhou, Guangxi, China). MS-grade acetonitrile, water, and formic acid were purchased from Merck (Billerica, MA, USA). Tamoxifen and cholic acid-d_4_ were obtained from Sigma-Aldrich Corporation (St. Louis, MO, USA). DSS was supplied by Meilun Biotechnology Co., Ltd. (Dalian, China). Reagents for chemical derivatization, including 4-acetamido-7-mercapto-2,1,3-benzoxadiazole (AABD-SH) and 2,2′-dipyridyl disulfide (DPDS), were purchased from TCI Development Co., Ltd. (Shanghai, China). Antibiotics (vancomycin, metronidazole, ampicillin, and neomycin sulfate) and organic acids/standards (triphenylphosphine (TPP), propionic acid, butyric acid, isovaleric acid, valeric acid, and hexanoic acid) were obtained from Macklin Biochemical Technology and Aladdin Biochemical Technology (Shanghai, China). Commercial kits for malondialdehyde (MDA) and glutathione (GSH) were purchased from Nanjing Jiancheng Bioengineering Institute (Nanjing, China). High-sensitivity ELISA kits for mouse TNF-α, IL-1β, and IL-6 were supplied by Jianglai Biotechnology (Shanghai, China).

### 2.2. Preparation of LBTE

LBT was pulverized into a fine powder and extracted with 70% ethanol at a solid-to-liquid ratio of 1:20 (*w*/*v*). The mixture was incubated in a 60 °C water bath for 2.5 h, then cooled to room temperature and filtered. The residue underwent two additional extractions under identical conditions. The combined filtrates were concentrated using a vacuum rotary evaporator at 60 °C and subsequently lyophilized to obtain the final LBTE powder.

### 2.3. Serum Pharmacochemistry Analysis

LBTE powder was dissolved in 70% methanol (1 mg/mL) and filtered through a 0.22 μm membrane for constituent profiling. For in vivo serum analysis, three mice were orally administered a high dose of LBTE (300 mg/kg). Blood samples were collected prior to dosing and 1 h post-administration. Serum was isolated, deproteinized with methanol, and centrifuged; the resulting supernatant was then evaporated to dryness. The residue was reconstituted in 70% methanol for LC-MS analysis. Analysis was performed using a Waters ACQUITY UPLC I-Class PLUS System coupled with a SELECT SERIES Cyclic IMS mass spectrometer (Waters, Milford, MA, USA). Chromatographic separation was achieved on an ACQUITY UPLC BEH C18 column (1.7 μm, 2.1 mm × 150 mm) following the parameters optimized in our previous study (detailed in Tables S1 and S2).

### 2.4. Network Pharmacology Analysis

Putative targets of LBTE constituents were predicted using SwissTargetPrediction (https://swisstargetprediction.ch/) (accessed on 13 October 2024), while UC-associated targets were retrieved from GeneCards (https://www.genecards.org/) (accessed on 17 October 2024). The intersection of these target sets was visualized via a Venn diagram. A protein–protein interaction (PPI) network was constructed using STRING 12.0 (https://string-db.org/) (accessed on 30 October 2024) and visualized in Cytoscape 3.9. Core targets were identified using the CytoHubba plug-in based on the Maximum Clique Centrality (MCC) algorithm. For functional enrichment analysis, the overlapping targets between LBTE-associated targets and UC-related targets were imported into Metascape (https://metascape.org/) (accessed on 30 October 2024), with Homo sapiens selected as the species. Gene Ontology (GO) enrichment analysis included biological process (BP), cellular component (CC), and molecular function (MF) categories, while Kyoto Encyclopedia of Genes and Genomes (KEGG) pathway enrichment analysis was performed to identify the major signaling and metabolic pathways associated with these targets. The analyses were conducted using the default parameters of Metascape, and significantly enriched terms were ranked according to their statistical significance for subsequent visualization.

### 2.5. Animals and Treatment

All animal procedures were approved by the Animal Ethics Committee of Guangxi University (Permit No: GXU-2024-320) and conducted in accordance with institutional guidelines. Seven-week-old male C57BL/6 mice (about 20 g) were obtained from Sibeifu Biotechnology (Beijing, China) and acclimated in a standardized environment with freely available access to food and sterile water during the experiment.

LBTE intervention study: Twenty-five mice were randomly assigned to five groups (*n* = 5): Normal Control (NC), Model Control (MC), Positive Control (PC; 5-aminosalicylic acid, 200 mg/kg), Low-dose LBTE (L, 100 mg/kg), and High-dose LBTE (H, 300 mg/kg). Groups received their respective treatments via oral gavage for 7 days. From day 8, UC was induced in all groups except the NC group by administering 3% DSS in drinking water for 7 consecutive days. During this 7-day DSS induction period, all treatments (5-aminosalicylic acid for PC, and LBTE for L and H groups) were continued daily via oral gavage. Body weight and disease activity index (DAI) were recorded daily. Meanwhile, daily fresh fecal samples from various groups were gathered for the later FMT study.

On day 14, corresponding to the end of the 7-day DSS administration period, mice were fasted for 12 h with free access to water. Blood was collected and serum was isolated by centrifugation. The mice were then euthanized, and fecal pellets, cecal contents, and the colon, spleen, heart, liver, lungs, and kidneys were harvested. The feces and cecal contents were snap-frozen immediately. The organs were rapidly excised, blotted dry, and weighed. A portion of the colon and organ tissues was fixed for histopathological analysis, while the remaining tissues and frozen fecal samples were aliquoted and stored at −80 °C for subsequent experiments. The organ indices of the spleen, heart, liver, lung, and kidney were all calculated using the following formula: Organ index (%) = (wet organ weight/final body weight of the mouse) × 100%.

Microbiota depletion (Abx) study: To assess microbiota dependence, mice were randomly divided into five experimental groups, including NC group, MC group, MC + Abx group (DSS + antibiotics), LBTE group, and LBTE + Abx group (LBTE + antibiotics). The Abx cocktail (including vancomycin (100 mg/kg), neomycin sulfate (200 mg/kg), metronidazole (200 mg/kg), and ampicillin (200 mg/kg)) was mixed and administered to the Abx groups for 7 days prior to and throughout the LBTE intervention.

Fecal microbiota transplantation (FMT) study: Recipient mice were depleted of endogenous microbiota using the Abx cocktail for 7 days. They were then randomly assigned to receive fecal suspensions from NC, MC, or LBTE-treated donors (FMT-NC, FMT-MC, and FMT-L, respectively) via daily oral gavage for 14 days, according to our established protocol [17].

### 2.6. ELISA and Biochemical Assays

Colon tissues were homogenized in ice-cold saline and centrifuged at 3500 rpm for 10 min at 4 °C. Protein concentrations were determined using a BCA assay. MDA and GSH levels in colon tissue and serum levels of TNF-α, IL-1β, and IL-6, were quantified using commercial kits following the manufacturers’ protocols.

### 2.7. Histopathological Evaluation

Colon and major organ samples were fixed in 4% paraformaldehyde, embedded in paraffin, and sectioned. Sections were stained with hematoxylin and eosin (H&E) for general morphology and periodic acid-Schiff (PAS) for goblet cell assessment. Observations were conducted under a light microscope (UOP, China).

### 2.8. 16S rRNA Microbiota Analysis

Cecal contents were collected and sequenced by Novogene Co., Ltd. (Beijing, China). The V4 region of the bacterial 16S rRNA gene was amplified using the forward primer (5′-GTGCCAGCMGCCGCGGTAA-3′) and reverse primer (5′-GGACTACHV GGGTWTCTAAT-3′), and then sequenced on the Illumina NovaSeq platform (Illumina, San Diego, CA, USA).

### 2.9. Quantitative Analysis of SCFAs

Fecal concentrations of SCFAs were determined using a modified derivatization- based LC-MS approach, as previously described [23]. Briefly, 50 mg of mouse cecal contents were suspended in 200 μL of ultrapure water and homogenized at 30 Hz for 1.5 min. The resulting homogenate was clarified through two consecutive rounds of centrifugation at 13,000 rpm (4 °C, 20 min) to harvest the supernatant. For chemical derivatization, the supernatant was reacted with 20 μL each of 20 mM AABD-SH, triphenylphosphine (TPP), and 2,2′-dipyridyl disulfide (DPDS) in dichloromethane. The reaction was facilitated by vortexing for 5 min at ambient temperature. Following derivatization, the mixture was lyophilized under vacuum and reconstituted in 40 μL of methanol containing tamoxifen (0.5 μM) as an internal standard. SCFA quantification was performed using external calibration curves established with standard solutions ranging from 10 nM to 1 mM. The LC-MS instrument settings and gradient conditions were consistent with our previously established protocols (detailed in Tables S3 and S4).

### 2.10. Untargeted Metabolomic Analysis

To ensure analytical robustness and detection accuracy, tamoxifen and cholic acid-d_4_ were utilized as internal standards. Serum samples were prepared following our previously established protocols with minor modifications. Quality control (QC) samples were generated by pooling equal aliquots of the supernatant from each individual specimen to monitor system stability and technical reproducibility throughout the analysis. Subsequently, untargeted metabolomics profiling was performed using a Waters ACQUITY UPLC I-Class PLUS System (Waters, Milford, MA, USA) (SN: L22BSP766G) coupled to a SELECT SERIES Cyclic IMS (Milford, MA, USA) (SN: GBC086). The liquid chromatography gradient conditions and mass spectrometry acquisition parameters were consistent with those employed for the serum pharmacochemistry analysis (detailed in Tables S1 and S2). Data were acquired in ESI+ and ESI− modes to ensure comprehensive metabolite coverage.

### 2.11. Integration of Metabolomics and Network Pharmacology

To elucidate the core targets and critical metabolic pathways underlying the therapeutic efficacy of LBTE against UC, integrated metabolomics and network pharmacology analyses were performed. A metabolites-reaction-enzyme-targets network was constructed by specifically importing differential metabolites characterized through metabolomic profiling into the Metscape plugin within Cytoscape 3.9. Key nodes were identified based on topological parameters; specifically, targets exceeding the median thresholds for degree, betweenness centrality (BC), and closeness centrality (CC) were prioritized. These high-value nodes were subsequently intersected with the core targets derived from disease databases to identify the pivotal pharmacological network.

### 2.12. RT-qPCR Analysis

Total RNA was isolated from colon tissues using TransZol Up (TransGen Biotech, Shanghai, China) and reverse-transcribed into cDNA using the SweScript All-in-One RT SuperMix (Servicebio, Wuhan, China). Quantitative PCR assays were conducted using SYBR Green qPCR Mix (Monad Biotech, Shanghai, China). The primer sequences utilized in this study were detailed in Table S5. Relative mRNA expression levels were normalized to *GAPDH* and quantified using the 2^−ΔΔCt^ method.

### 2.13. Western Blot Analysis

Colon tissues were homogenized on ice in RIPA lysis buffer supplemented with protease inhibitors for 5–10 min. The homogenate was centrifuged at 12,000 rpm for 10 min at 4 °C, and the protein-containing supernatant was harvested. Protein concentrations were normalized, and samples were mixed with loading buffer and denatured at 95 °C for 10 min. Equivalent amounts of protein samples were separated by 7.5% SDS-PAGE and transferred onto PVDF membranes. After blocking for 2 h at room temperature, membranes were incubated overnight at 4 °C with primary antibodies against ZO-1, occludin, ALOX5, PTGS1, PTGS2, P65, p-P65, CYP3A11 and CYP19A1 (diluted 1:10,000). Subsequently, the membranes were incubated with HRP-conjugated secondary antibodies for 1.5 h. Proteins were visualized using an ECL in a chemiluminescence imaging system, and densitometric analysis was performed using ImageJ 1.53k software (National Institutes of Health, USA).

### 2.14. Data Processing and Statistical Analysis

The raw multi-dimensional data derived from untargeted metabolomics was systematically deconvoluted using Progenesis QI 2.0 software (Waters, Milford, MA, USA) for peak alignment, normalization, and feature extraction. To ensure data quality, peak intensities were dynamically normalized to quality control (QC) samples. Metabolite identification was performed by cross-matching the accurate mass, isotopic patterns, and tandem MS/MS fragments against the METLIN, LipidBlast, and Human Metabolome Database (HMDB). Supervised and unsupervised multivariate statistical analyses, including Principal Component Analysis (PCA) and Orthogonal Partial Least Squares Discriminant Analysis (OPLS-DA), were conducted using SIMCA-P 14.0 software (Umetrics, Umea, Sweden). The robustness of the OPLS-DA models was rigorously cross-validated invoking a 200-time permutation test to preclude mathematical overfitting.

Univariate statistical comparisons were performed utilizing Microsoft Excel and GraphPad Prism 8. For pairwise comparisons, a two-tailed Student’s *t*-test was employed. All data were presented as the mean ± standard deviation (SD). A critical threshold of *p* < 0.05 was designated as the definitive baseline for statistical significance. Metabolic pathway enrichment analysis was functionally interrogated invoking the MetaboAnalyst 6.0 platform. Multi-omics integration linking discrete individual metabolic profiles with host clinical phenotypic vectors was mathematically established via two-dimensional Spearman correlation coordinate analysis.

## 3. Results and Discussions

### 3.1. Identification of Absorbable Phytochemicals by Serum Pharmacochemistry

To elucidate the potential bioactive constituents of LBTE, comprehensive serum chemical profiling was performed following oral administration. Absorbed prototype compounds were characterized by integrating retention times, accurate mass measurements, and characteristic secondary fragment ions, and cross-referencing with commercial databases (Table S6). Based on LC-MS-based serum pharmacokinetic profiles, a total of 11 prototype compounds were detected in serum samples, including 4 terpenoids, 2 organic acids, 1 flavonoid, 1 alkaloid, 1 glycoside, 1 coumarin, and 1 lignan (Figure S1A–F). These identified phytochemicals are consistent with previously reported profiles of LBTE [16]. Notably, several identified constituents have well-documented pharmacological activities relevant to intestinal homeostasis. For instance, triptolide has been demonstrated to exert potent anti-UC effects by inhibiting macrophage infiltration, modulating M1-type polarization, and suppressing the secretion of pro-inflammatory cytokines [24]. Similarly, epigallocatechin has been reported to alleviate UC by forming a protective bio-nanocoating that reshapes the gut microbiota and restores intestinal barrier integrity [25]. These absorbable compounds were subsequently prioritized as candidate ligands for the integrated network pharmacology analysis to further disclose LBTE’s multi-target mechanisms.

### 3.2. Network Pharmacology Analysis Based on Absorbed Phytochemicals

**Figure 1:**
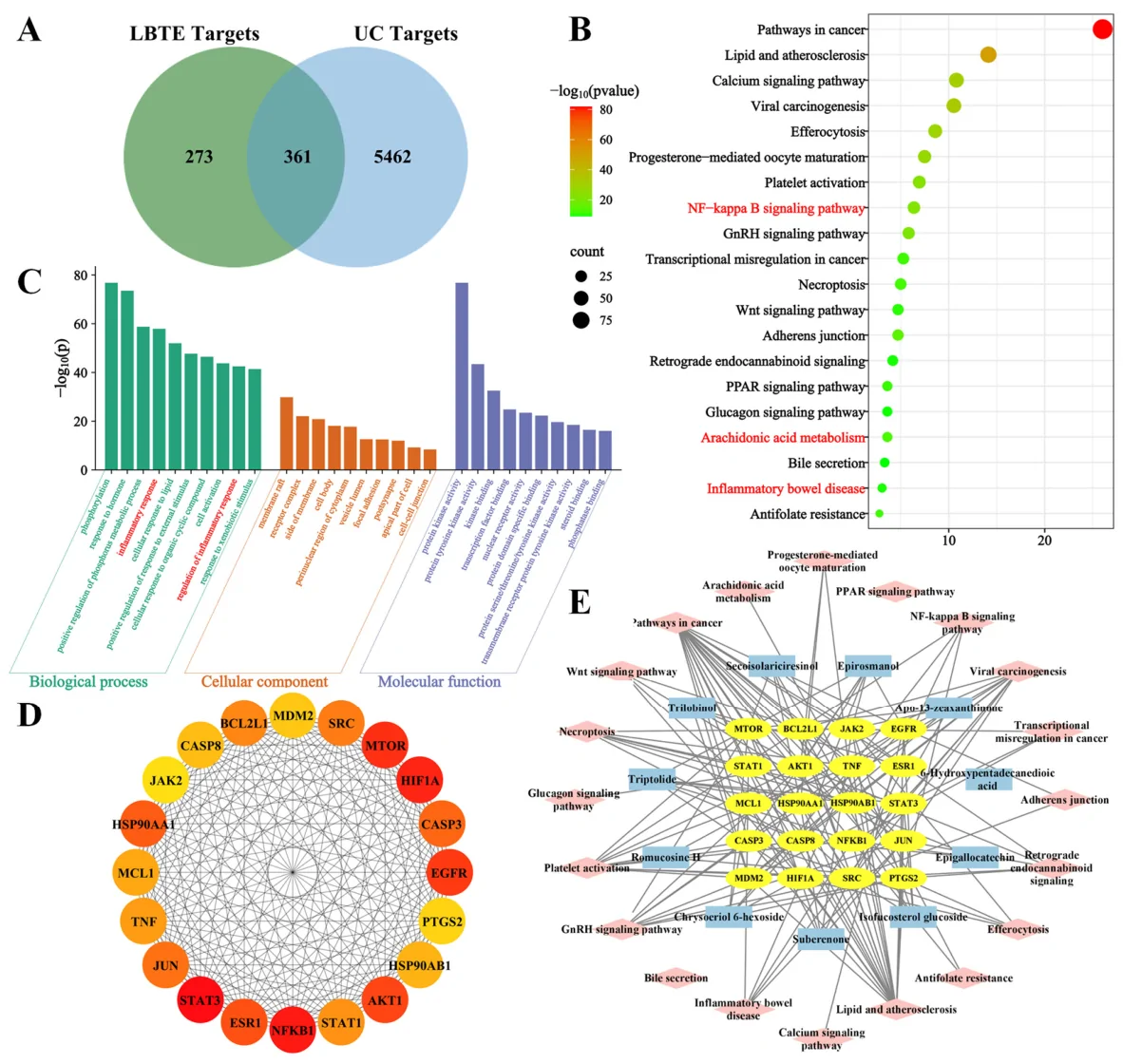
Integrative network pharmacology analysis identifying potential therapeutic targets of LBTE against UC. (**A**) Venn diagram illustrating overlapping genes between LBTE-associated targets and UC-related pathogenic genes. (**B**) KEGG enrichment analysis highlighting the top 20 signaling pathways regulated by LBTE. (**C**) GO enrichment analysis identifying the top 10 biological processes. (**D**) Topology-based identification of the top 20 candidate targets. (**E**) Ingredient-target-pathway interaction network.

The potential bioactive constituents of LBTE identified via serum pharmacochemistry were subjected to network pharmacology investigation, yielding 634 putative compound-associated targets. Concurrent mining of disease-related databases retrieved 5823 UC-associated genes. A Venn diagram analysis identified 361 overlapping targets (Figure 1A), which were defined as the potential therapeutic nodes of LBTE against UC. To elucidate the functional landscape of these shared targets, KEGG pathway enrichment analysis was performed. The results revealed significant enrichment in the NF-κB signaling pathway, arachidonic acid metabolism, and inflammatory bowel disease-related pathways (Figure 1B), all of which are closely associated with UC pathogenesis. Specifically, inhibition of the NF-κB signaling cascade has been shown to alleviate colonic inflammation by modulating the expression of downstream pro-inflammatory cytokines [26,27]. Moreover, aberrant arachidonic acid metabolism and the subsequent accumulation of eicosanoid metabolites are hallmark features of colonic mucosal injury in UC patients [28]. Complementary GO enrichment analysis further confirmed the involvement of these targets in biological processes predominantly related to inflammatory response and immune regulation (Figure 1C). To pinpoint the core therapeutic nodes, a protein–protein interaction (PPI) network was constructed and subjected to topology analysis. The top 20 hub genes were prioritized based on their centrality scores (Figure 1D). Ultimately, a comprehensive “compound–target–pathway” network was developed to demonstrate that LBTE exerts its therapeutic effects on UC by a synergistic, multi-component, and multi-pathway regulatory mechanism, with a particular emphasis on restoring inflammatory and metabolic homeostasis (Figure 1E).

### 3.3. LBTE Alleviates the Systemic Inflammation and Disease Phenotypes of DSS-Induced UC Mice

**Figure 2:**
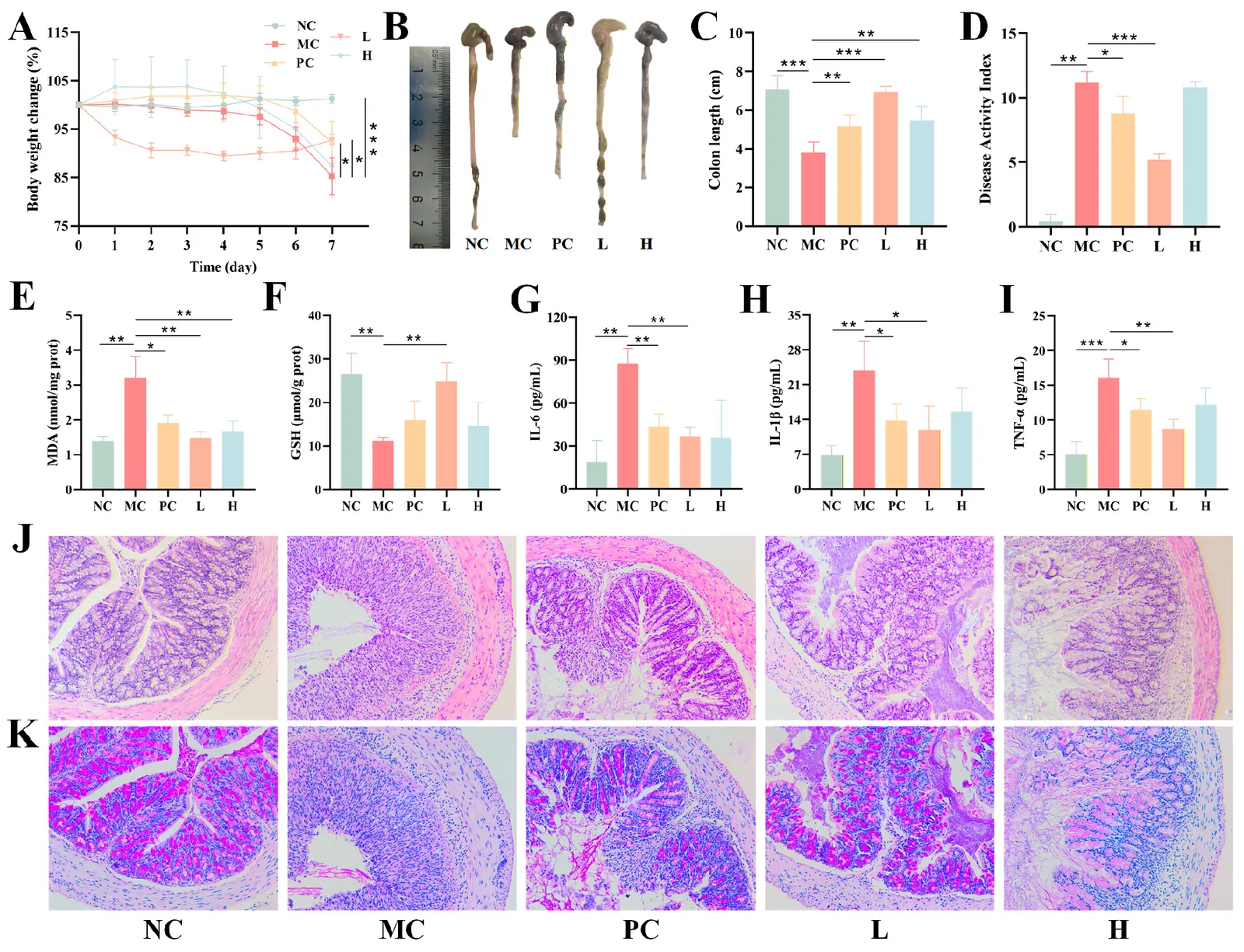
LBTE attenuates clinical symptoms, systemic inflammation, and colonic histopathology in DSS-induced UC mice. (**A**) Body weight changes during DSS induction. (**B**,**C**) Representative images and statistical quantification of colon length. (**D**) Daily disease activity index (DAI) scores. (**E**) Malondialdehyde (MDA) levels. (**F**) Glutathione (GSH) levels. (**G**–**I**) levels of pro-inflammatory cytokines: IL-6, IL-1β, and TNF-α. (**J**,**K**) Representative micrographs of H&E and PAS-stained colon sections (200× magnification). Data are expressed as mean ± SD. * *p* < 0.05, ** *p* < 0.01, *** *p* < 0.001 vs. the MC group. NC: Normal Control, MC: Model Control, PC: Positive Control, L: Low-dose LBTE, H: High-dose LBTE.

To evaluate the therapeutic efficacy of LBTE, a DSS-induced mouse model of acute colitis was employed. Clinical severity was systematically quantified using the Disease Activity Index (DAI), which integrates body weight fluctuations, stool consistency, and gross bleeding. Compared to the NC group, mice in the MC group exhibited hallmark clinical manifestations of colitis, including profound body weight loss, significant colonic shortening, elevated DAI scores, and an increased spleen index, confirming the successful establishment of the UC model (Figure 2A–D and Figure S2A; *p* < 0.05). Histopathological examination of splenic tissue from the MC group revealed severe structural disorganization, characterized by the destruction of white pulp and blurred marginal zones, indicative of systemic immune dysregulation (Figure S2H). In contrast, intervention with either the positive control drug or LBTE (particularly in the L and PC groups) markedly attenuated these clinical and pathological parameters (*p* < 0.05). Notably, while terpenoids such as triptolide possess potent anti-inflammatory properties, their narrow therapeutic window and associated hepatotoxicity may account for the diminished efficacy observed in the high-dose (H) group [29]. Importantly, aside from this dose-dependent response, no histopathological lesions or adverse effects were observed in other major organs, supporting the overall safety profile of LBTE at effective therapeutic doses (Figure S2B–G,I,J).

Oxidative stress, a critical driver of UC pathogenesis, was significantly pronounced in the MC group, as evidenced by a substantial increase in MDA levels and a concomitant depletion of GSH (Figure 2E,F; *p* < 0.05). This redox imbalance was effectively reversed by treatment with positive control drug or LBTE. Concurrently, levels of pro-inflammatory cytokines (TNF-α, IL-1β, and IL-6) were significantly suppressed following LBTE administration, with the low-dose group demonstrating the most robust anti-inflammatory response (Figure 2G–I; *p* < 0.05). Histopathological profiling further corroborated the therapeutic effects of LBTE against UC. The extensive mucosal erosion, crypt architectural distortion, and massive inflammatory infiltration observed in the MC group were substantially rectified by LBTE intervention (Figure 2J). Furthermore, PAS staining highlighted that LBTE preserved the mucin layer thickness and goblet cell density, which are essential for maintaining the biological barrier against luminal insults (Figure 2K). Overall, these findings suggest that LBTE confers multi-dimensional protection against DSS-induced UC by harmonizing redox homeostasis, suppressing cytokine storms, and fortifying the intestinal epithelial barrier.

### 3.4. LBTE Ameliorates Gut Microbiota Dysbiosis and Remodels Microbial Architecture in UC Mice

**Figure 3:**
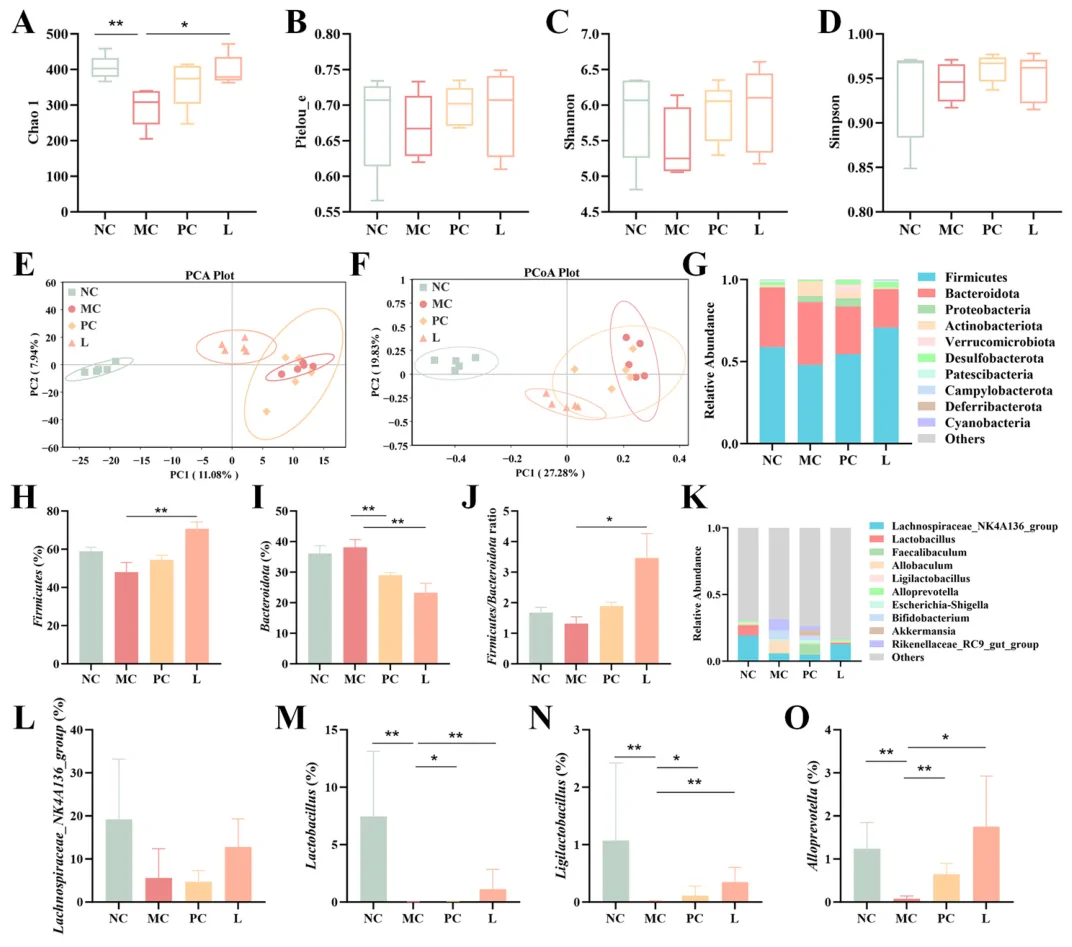
LBTE reshapes gut microbiota profiles in UC mice. (**A**–**D**) α-diversity indices. (**E**,**F**) β-diversity visualized by PCA and PCoA plots at the OTU level. (**G**) Relative abundance of gut microbiota at phylum level. (**H**) *Firmicutes* abundance, (**I**) *Bacteroidota* abundance, and (**J**) *Firmicutes/Bacteroidota* ratio. (**K**) Relative abundance of gut microbiota at genus level. (**L**–**O**) Abundance of beneficial SCFA-producing genera: *Lachnospiraceae_NK4A136_group*, *Lactobacillus*, *Ligilactobacillus*, and *Alloprevotella*. Data are presented as mean ± SD. * *p* < 0.05, ** *p* < 0.01 vs. the MC group.

Gut microbiota dysbiosis is a hallmark of UC, characterized by a loss of microbial diversity and the expansion of pathobionts. To evaluate the restorative effects of LBTE on the enteric ecosystem, we performed 16S rRNA gene sequencing on cecal contents. α- and β-diversity indices served as indicators for the diversity of the gut microbiota. Based on Chao 1, Pielou_e, Shannon and Simpson indices, α-diversity analysis demonstrated that DSS administration reduced the richness and evenness of the gut microbiota, whereas LBTE treatment partially reversed these parameters, bolstering the overall diversity of the microbial community (Figure 3A–D). To assess inter-group phylogenetic similarities, β-diversity was evaluated using multivariate statistical analysis including principal component analysis (PCA) and principal coordinate analysis (PCoA). Both ordination plots revealed a distinct clustering of the MC group away from the NC group, whereas the LBTE-treated groups exhibited a clear trajectory back toward the NC cluster (Figure 3E,F). These results indicate that, at the macroecological level, the LBTE-mediated alleviation of UC is closely linked to the restoration of microbial ecosystem stability. However, the therapeutic efficacy of LBTE is mechanistically anchored in the selective remodeling of specific functional taxa.

At the phylum level, the MC group exhibited a characteristic dysbiosis signature, marked by a contraction of Firmicutes and a concomitant expansion of Bacteroidota. LBTE administration effectively reversed these shifts in UC mice (Figure 3G–I). Aligning with previous investigations, the *Firmicutes/Bacteroidota* ratio, a critical marker for microbial equilibrium, was diminished in UC mice but was robustly restored following LBTE treatment (Figure 3J). This recovery signifies a transition toward an anti-inflammatory microbial environment, as the *Firmicutes* encompass numerous beneficial taxa involved in SCFA production [30]. Further genus-level profiling revealed that LBTE reshaped the microbial landscape by suppressing potential pathogens and enriching symbiotic taxa (Figure 3K–O). In the MC group, the relative abundance of the intestinal pathogen *Escherichia–Shigella* was markedly increased, whereas beneficial genera *Lactobacillus*, *Ligilactobacillus*, and *Alloprevotella* were significantly decreased [31,32]. Importantly, LBTE treatment not only effectively reversed these alterations but also specifically enriched SCFA-producing genera, including *Lachnospiraceae_NK4A136_group*, *Lactobacillus*, *Ligilactobacillus*, and *Alloprevotella*. These taxa are essential for maintaining epithelial barrier integrity and orchestrating mucosal immune homeostasis through the production of metabolic byproducts [33,34]. Collectively, these findings underscore that LBTE exerts its therapeutic effects by remodeling the gut microbiota and promoting a diverse, homeostatic community structure.

### 3.5. LBTE Promotes SCFA Production and Enhances Intestinal Barrier Function

**Figure 4:**
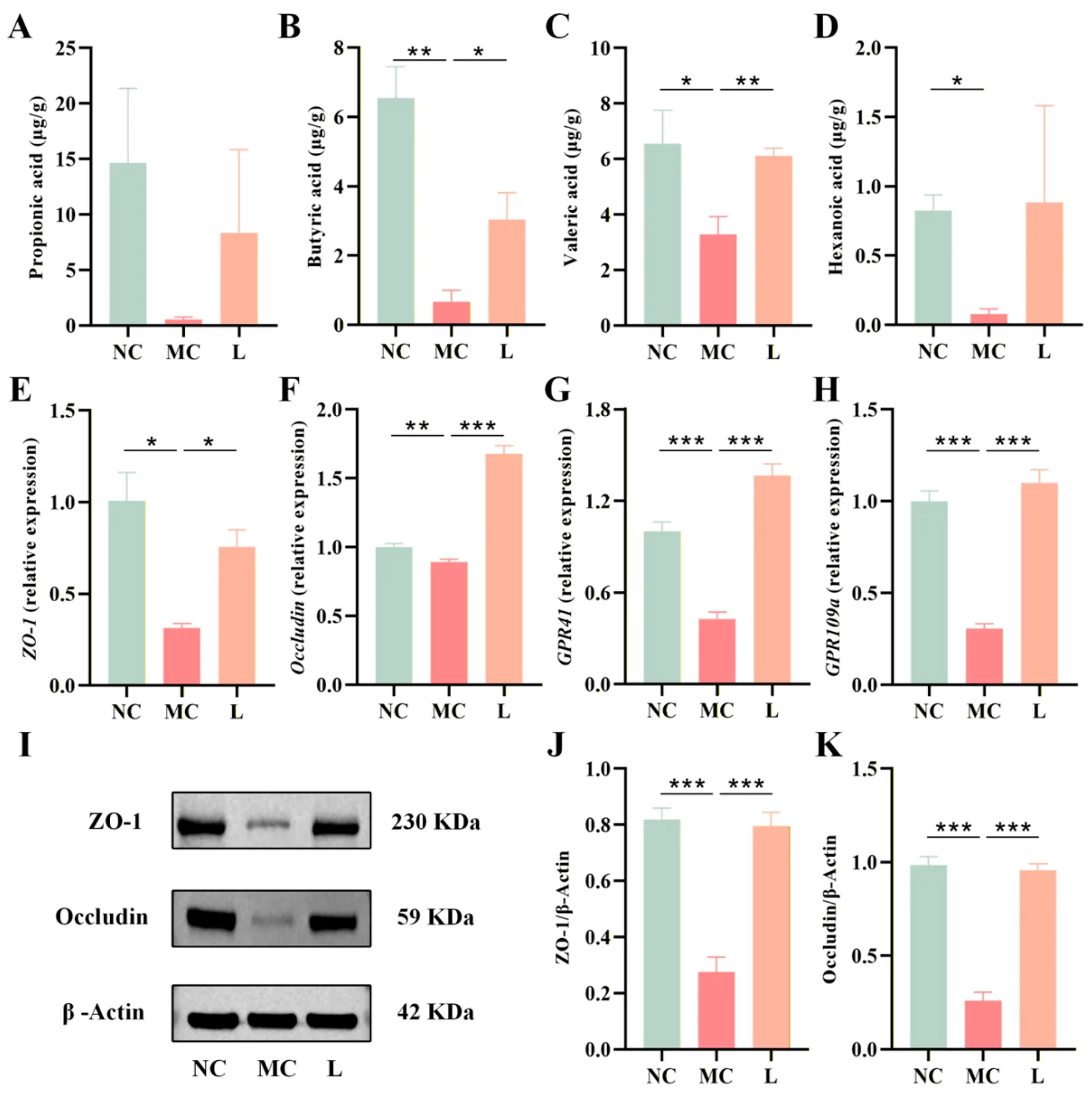
LBTE restores intestinal barrier function and SCFA-related signaling in UC mice. (**A**–**D**) Concentration of SCFAs, including propionic acid, butyric acid, valeric acid, and hexanoic acid from cecal contents. (**E**,**F**) Relative mRNA expression of tight junction proteins (*ZO-1* and *occludin*) in the colon. (**G**,**H**) Relative mRNA expression of SCFA-responsive receptors (*GPR41* and *GPR109a*) in the colon. (**I**–**K**) Protein expression levels of ZO-1 and occludin in the colon. Data are displayed as mean ± SD. * *p* < 0.05, ** *p* < 0.01, *** *p* < 0.001 vs. the MC group.

The intestinal barrier serves as a critical therapeutic target in UC, where its structural integrity and homeostatic regulation are profoundly influenced by SCFAs, the key byproducts from gut microbiota fermentation [19]. Earlier research has indicated that LBTE restores microbial balance and enriches SCFA-producing taxa. To further clarify the role of LBTE in barrier protection, SCFA profiles in the cecal contents were quantified. Compared to the NC group, a significant reduction in total and individual SCFA concentrations was observed in UC mice (Figure 4A–D). Notably, LBTE administration effectively counteracted this decline, specifically leading to a robust elevation in butyric acid and valeric acid levels compared to the MC group (*p* < 0.05). SCFAs are known to reinforce the intestinal epithelial barrier by upregulating the expression of tight junction proteins, such as ZO-1 and occludin [35]. Consistently, the Western blot and RT-qPCR analyses revealed that the colonic expression of ZO-1 and occludin was significantly suppressed in the MC group. However, this downregulation was markedly reversed by low-dose LBTE treatment (Figure 4E,F,I–K; *p* < 0.05). To further dissect the signaling pathways involved in SCFA-mediated effects, the expression of GPR41 and GPR109a, two key G-protein-coupled receptors (GPCRs) that function as critical downstream mediators of SCFA signaling, was examined. These receptors are pivotally involved in promoting mucosal healing and suppressing colonic inflammation, making them emerging therapeutic targets in IBD [36,37]. In the present study, the expression of both GPR41 and GPR109a was markedly downregulated in the MC group but was significantly restored following low-dose LBTE intervention (Figure 4G,H; *p* < 0.001). Consequently, these results indicate that LBTE facilitates the biosynthesis of gut microbiota-derived SCFAs, which in turn reinforces intestinal barrier integrity by promoting the expression of tight junction proteins and activating SCFA-responsive receptors. These molecular changes are in high alignment with the observed histopathological recovery in DSS-induced mice.

### 3.6. LBTE Modulates Arachidonic Acid Metabolism in UC Mice

**Figure 5:**
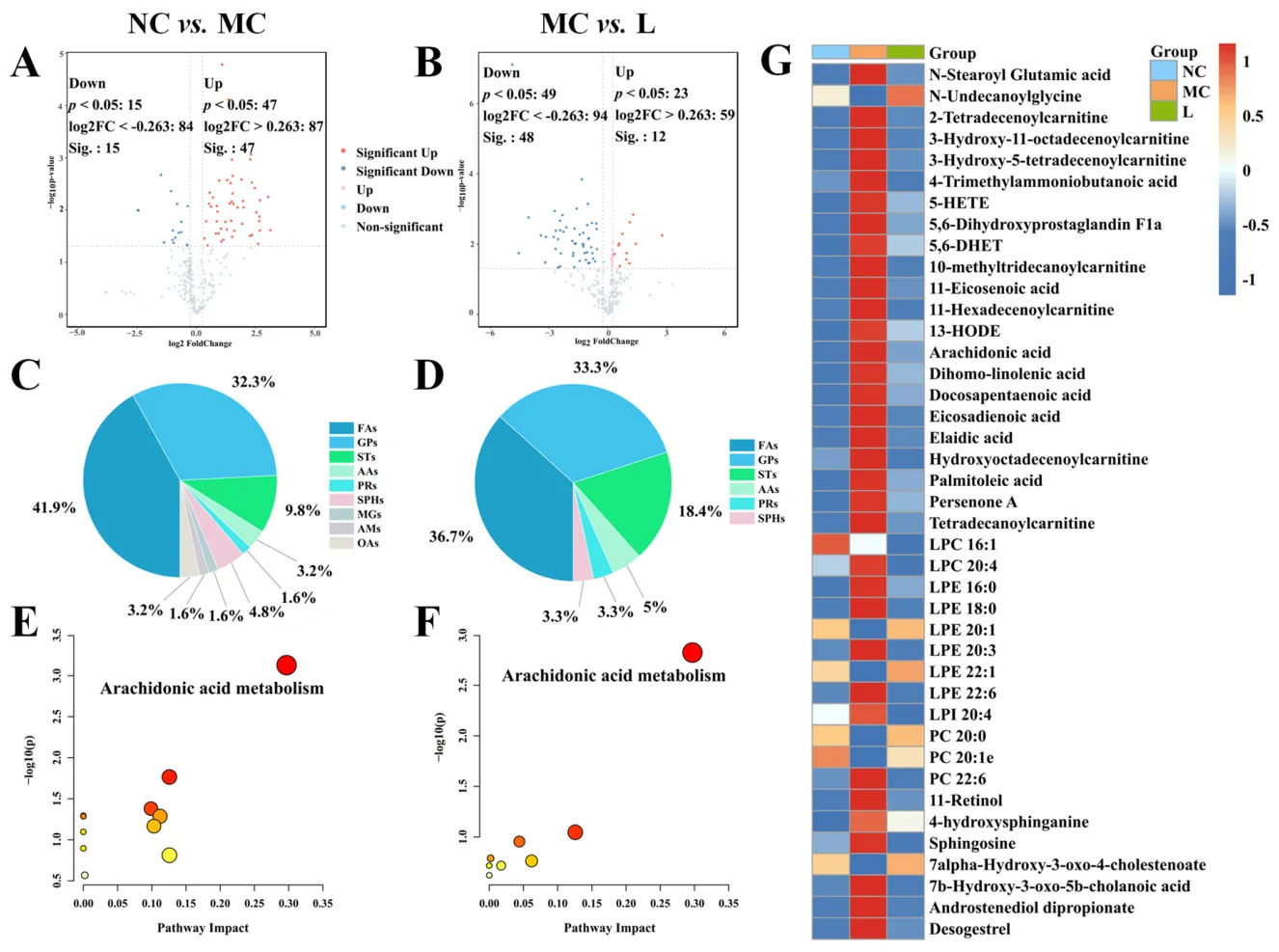
Effects of LBTE on serum metabolic profiles in UC mice. (**A**,**B**) Volcano plots illustrating differential metabolites between the NC vs. MC and MC vs. L groups. (**C**,**D**) Chemical classification and constituent ratios of differential metabolites. (**E**,**F**) Pathway enrichment analysis of differential metabolites. (**G**) Heatmap analysis of pivotal discriminatory metabolites across the three experimental groups.

To elucidate the metabolic perturbations associated with UC and the compensatory effects of LBTE, untargeted serum metabolomic profiling was performed using a UPLC-Q-TOF-MS platform. The tight clustering of quality control (QC) samples in the PLS-DA score plot confirmed the robust stability and high reproducibility of the analytical system (Figure S3A,B). OPLS-DA further illustrated distinct metabolic differences among the various experimental groups, with clear clustering and separation visible in the score plots (Figure S3C,D). While the distinct separation was evident between the NC and MC groups, the L-dose LBTE group displayed a profile that trended back toward the NC group, indicating that LBTE effectively neutralized DSS-induced systemic metabolic disruptions. The robustness of these models was validated by 200 permutation tests, with *R*^2^*Y* and *Q*^2^ values affirming the absence of overfitting in ESI+ and ESI- modes (Figure S3E,F). Differential metabolites were screened according to the criteria of fold change (FC) >1.2 or <0.83 and *p* < 0.05, and visualized via volcano plots. Between the MC and NC groups, 47 metabolites were upregulated and 15 were downregulated (Figure 5A). Between the L and MC groups, 12 metabolites were upregulated and 48 were downregulated (Figure 5B). Fatty acids (FAs), glycerophospholipids (GPs), and steroids (STs) were the main classes for the differential metabolites (Figure 5C,D). Notably, the MC group demonstrated a substantial accumulation of pro-inflammatory lipid mediators, such as arachidonic acid, 5-hydroxyeicosatetraenoic acid (5-HETE), 5,6-dihydroxyprostaglandin F1α, and 5,6-dihydroxy-eicosatrienoic acid (5,6-DHET), all of which were significantly decreased after LBTE treatment (Figure 5G and Figure S4). Arachidonic acid acts as a critical precursor of pro-inflammatory lipid-derived mediators and undergoes metabolism via diverse enzymatic pathways to generate bioactive metabolites [38]. Significantly, 5-HETE is a primary product of the 5-lipoxygenase (5-LOX) pathway, playing a crucial role in leukocyte recruitment and pro-inflammatory signaling [39]. Simultaneously, 5,6-dihydroxyprostaglandin F1α is produced through the COX pathway and reflects intensified prostaglandin-mediated inflammatory responses [40]. Additionally, the elevation of 5,6-DHET, the hydrolysis product of CYP450-derived epoxyeicosatrienoic acids (EETs) via soluble epoxide hydrolase (sEH), represents pathological activation of the CYP450/sEH branch of arachidonic acid metabolism [41]. The collective surge of these arachidonic acid-derived pro-inflammatory metabolites in UC pathogenesis signified a profound dysregulation of the arachidonic acid metabolic network, whereas their reduction following LBTE intervention suggests that LBTE may mitigate inflammation by modulating this metabolic pathway. Pathway enrichment analysis corroborated that arachidonic acid metabolism was the most significantly disrupted pathway in UC and served as a primary target for LBTE-mediated modulation (Figure 5E,F). Collectively, these findings suggest that LBTE ameliorates UC-associated inflammation by broadly suppressing multiple enzymatic branches of the arachidonic acid cascade, leading to restoration of lipid metabolic homeostasis.

### 3.7. Screening and Validation of Key Targets in the Arachidonic Acid Metabolism

**Figure 6:**
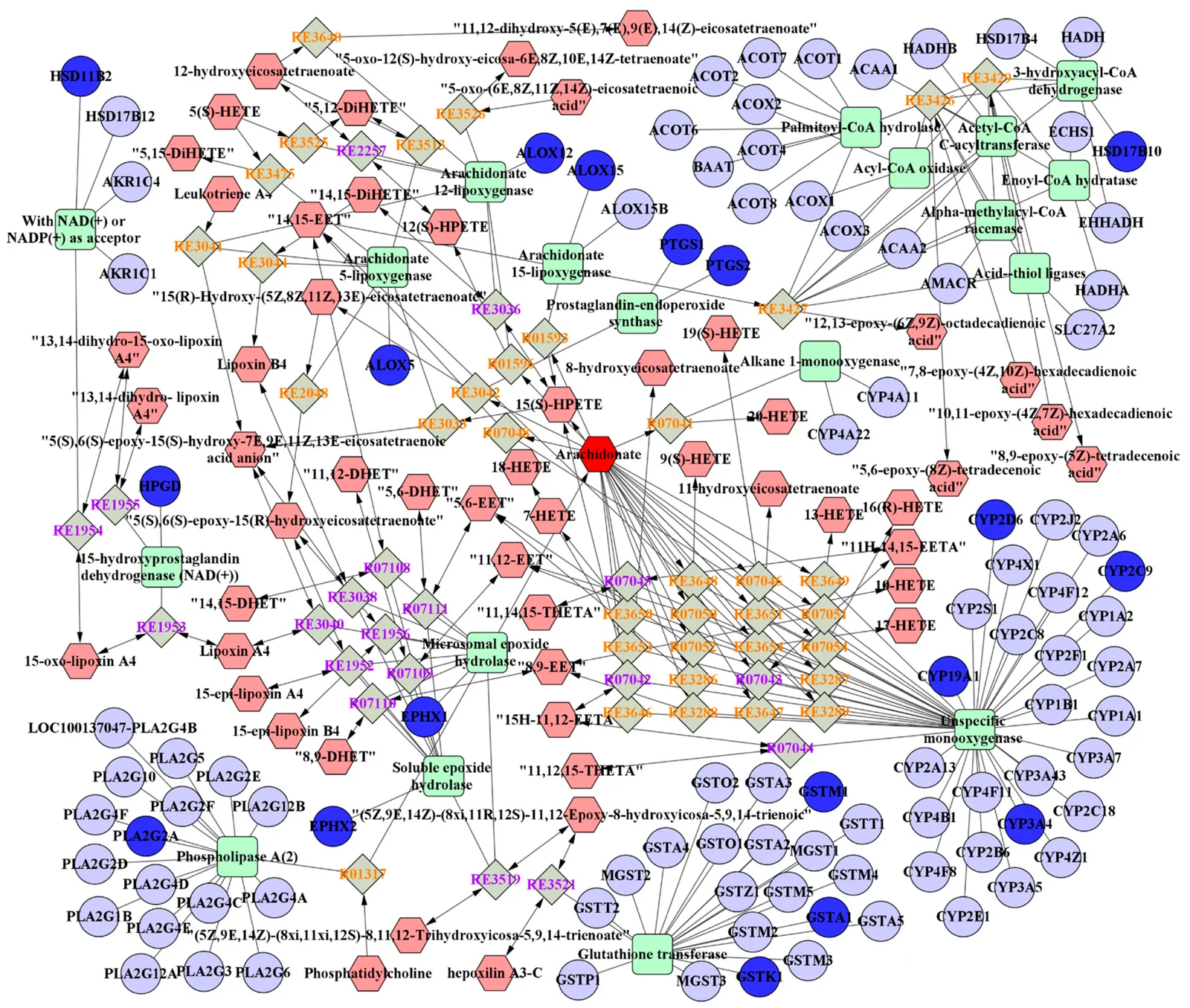
Screening of core targets involved in arachidonic acid metabolic pathway. Compound-reaction-enzyme-gene networks for differential metabolites. In the network, different nodes represent metabolites (red), active compounds (pink), reactions (light green), and key targets (blue), respectively.

**Figure 7:**
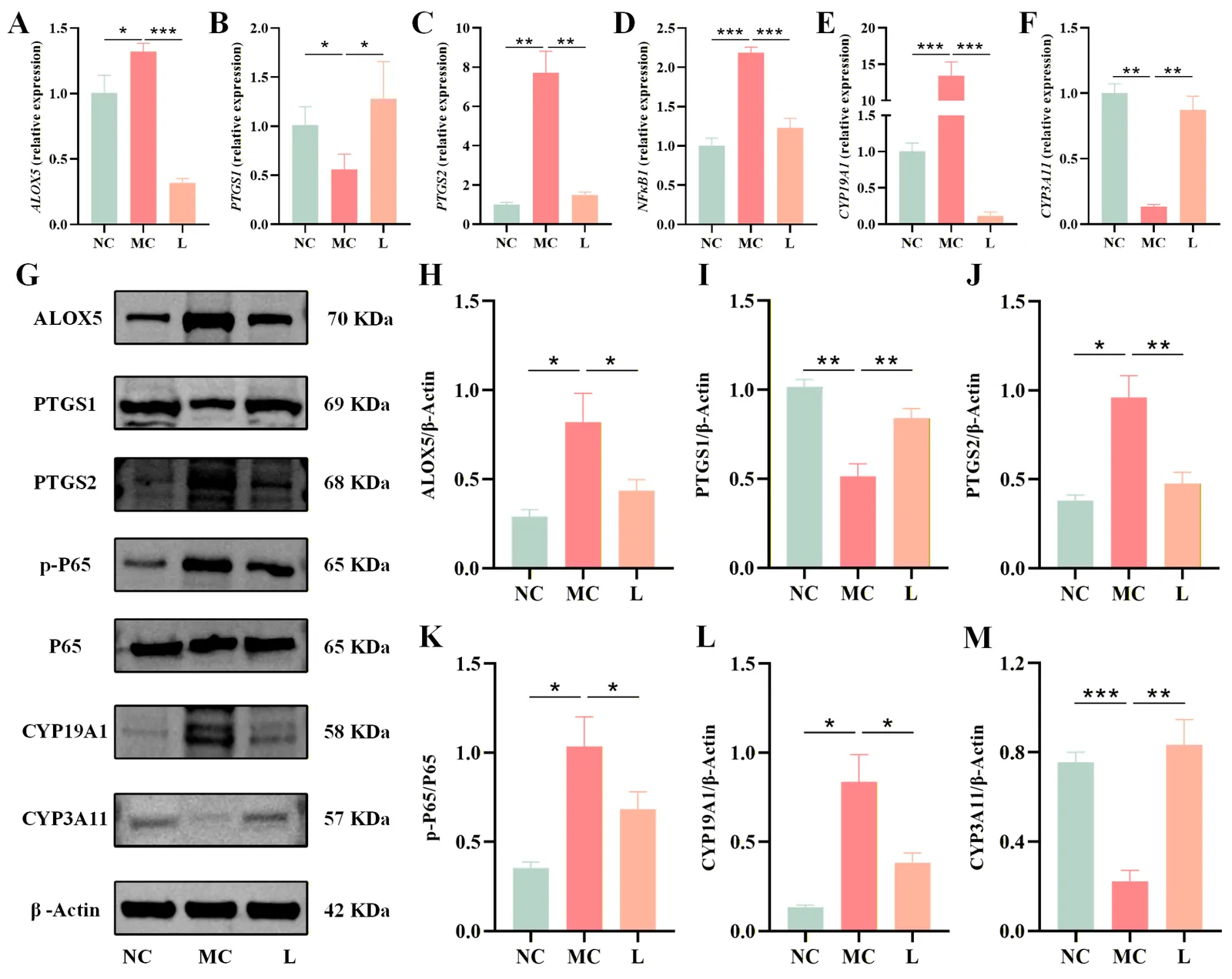
Validation of core targets involved in arachidonic acid metabolic pathway. (**A**–**F**) mRNA expression levels of *ALOX5*, *PTGS1*, *PTGS2*, *NFκB1*, *CYP19A1*, and *CYP3A11*. (**G**–**M**) Protein expression levels of ALOX5, PTGS1, PTGS2, p-P65/P65, CYP3A11, and CYP19A1 in the colon. Data are displayed as mean ± SD. * *p* < 0.05, ** *p* < 0.01, *** *p* < 0.001 vs. the MC group.

To further elucidate the molecular mechanisms underlying LBTE’s anti-inflammatory effects, a comprehensive “metabolites-reaction-enzyme-gene” network was constructed in Cytoscape 3.9.1 by integrating differential metabolites with network pharmacology-derived targets. This network revealed core regulatory nodes closely associated with arachidonic acid metabolism. As shown in Figure 6, topological analysis of this integrated network identified five core regulatory nodes (*PTGS1*, *PTGS2*, *ALOX5*, *CYP3A11*, and *CYP19A1*) as pivotal mediators of LBTE’s therapeutic efficacy in UC. Experimental validation via RT-qPCR and Western blot confirmed the regulatory impact of LBTE on these targets. PTGS2 (COX-2), an inducible cyclooxygenase that catalyzes the conversion of arachidonic acid into pro-inflammatory prostaglandins, is typically overexpressed in colonic mucosa during active inflammation [42]. By contrast, PTGS1 (COX-1), a constitutive isoform crucial for mucosal protection, is frequently suppressed during colitis [43]. The results demonstrated that LBTE treatment evidently suppressed PTGS2 expression while robustly restoring PTGS1 levels (Figure 7B,C,I–J; *p* < 0.05), suggesting that LBTE inhibits inflammatory prostaglandin synthesis while preserving epithelial integrity. Furthermore, LBTE effectively downregulated ALOX5 (5-LOX), a key enzyme driving the production of leukotriene B4 and subsequent mucosal inflammatory cell infiltration [44,45], which was markedly elevated in the MC group (Figure 7A,H; *p* < 0.05). Moreover, LBTE normalized the expression of CYP3A11 and CYP19A1 (Figure 7E,F,L,M; *p* < 0.05), two key cytochrome P450 enzymes involved in the oxidative metabolism and homeostatic clearance of arachidonic acid derivatives [46]. Notably, LBTE also inhibited the expression of NFκB1 (Figure 7D,K; *p* < 0.05), a master inflammatory transcription factor that activates PTGS2 and ALOX5, thereby attenuating UC-associated inflammatory cascades [47]. Therefore, these findings suggest that LBTE mitigates UC-associated inflammatory cascades through the coordinated modulation of the arachidonic acid metabolic pathway. By suppressing NFκB1, PTGS2, and ALOX5, while restoring protective metabolic enzymes, such as PTGS1 and CYP3A11, LBTE reestablishes arachidonic acid metabolic homeostasis and reduces the production of inflammatory eicosanoids, highlighting its potential as a multi-target therapeutic strategy for inflammatory and metabolic disorders.

### 3.8. Integrative Correlation Analysis of Gut Microbiota Topology, Metabolic Profiles, and Clinical Parameters

To further elucidate the systemic interplay between LBTE-modulated gut microbiota, host metabolism, and UC pathogenesis, a Spearman correlation analysis was performed across genus-level microbial taxa, differential metabolites, and clinical indices. In Figure S5, beneficial genera, specifically *Lactobacillus* and *Ligilactobacillus*, exhibited robust positive correlations with body weight, colon length, and GSH levels, while displaying strong negative correlations with DAI, pro-inflammatory cytokines (IL-1β, TNF-α, IL-6), and MDA levels. These associations reinforce their putative role as key microbial mediators in the therapeutic efficacy of LBTE. Conversely, the *Rikenellaceae_RC9_gut_group* exhibited a diametrically opposite correlation pattern with these UC-related clinical-pathological factors, suggesting its potential contribution to the exacerbation of colonic injury. Furthermore, integrative analysis revealed a significant metabolic-microbial link. Arachidonic acid and its pro-inflammatory derivatives (5-HETE, 5,6-Dihydroxyprostaglandin F1α, and 5,6-DHET) were positively associated with the abundance of potential pathobionts, such as *Escherichia-Shigella*, *Bifidobacterium*, and *Rikenellaceae_RC9_gut_group* (*p* < 0.05). In contrast, these inflammatory lipid mediators were inversely correlated with the enrichment of *Lactobacillus* and *Ligilactobacillus* (Figures S6 and S7; *p* < 0.05). Interestingly, although *Bifidobacterium* is often regarded as beneficial, its specific correlation profiles in this context warranted further scrutiny, potentially reflecting a complex compensatory response during dysbiosis. Collectively, these multidimensional correlations indicate that specific bacterial genera, particularly *Lachnospiraceae_NK4A136_group*, *Lactobacillus*, and *Ligilactobacillus*, may serve as pivotal microbial biomarkers that dictate the therapeutic effects of LBTE. These taxa likely mediate the anti-UC effects of LBTE by coordinately modulating arachidonic acid and SCFA metabolism, thereby restoring systemic and local intestinal homeostasis.

### 3.9. Anti-UC Effects Mediated by LBTE Mainly Depend on the Gut Microbiota

**Figure 8:**
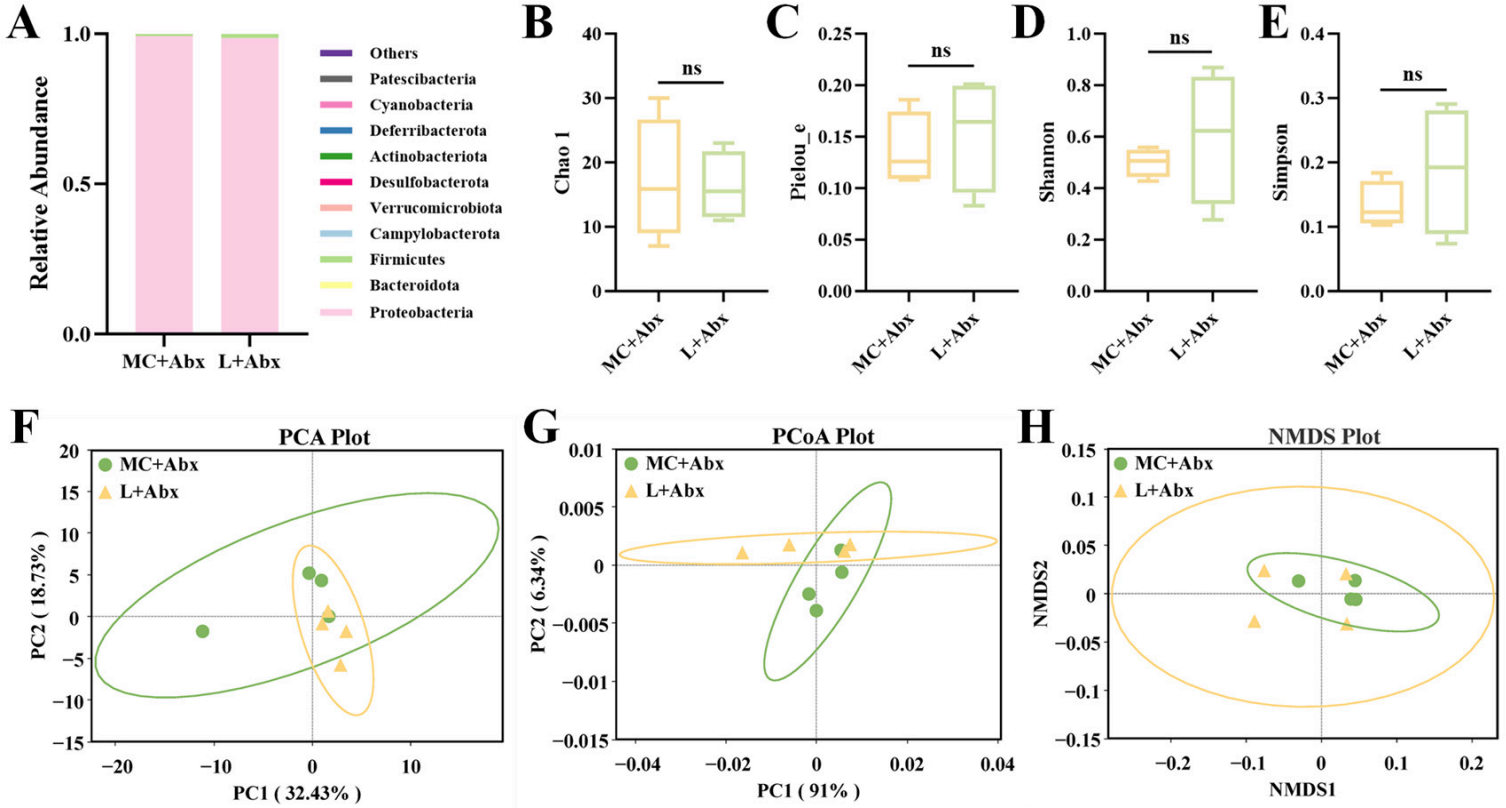
Validation of pseudo-germ-free (PGF) mouse model by 16S rRNA sequencing. (**A**–**H**) Antibiotic-induced gut microbiota depletion was demonstrated by phylum-level distribution, α-diversity, and β-diversity analyses. Data are presented as mean ± SD. ns: *p* > 0.05.

**Figure 9:**
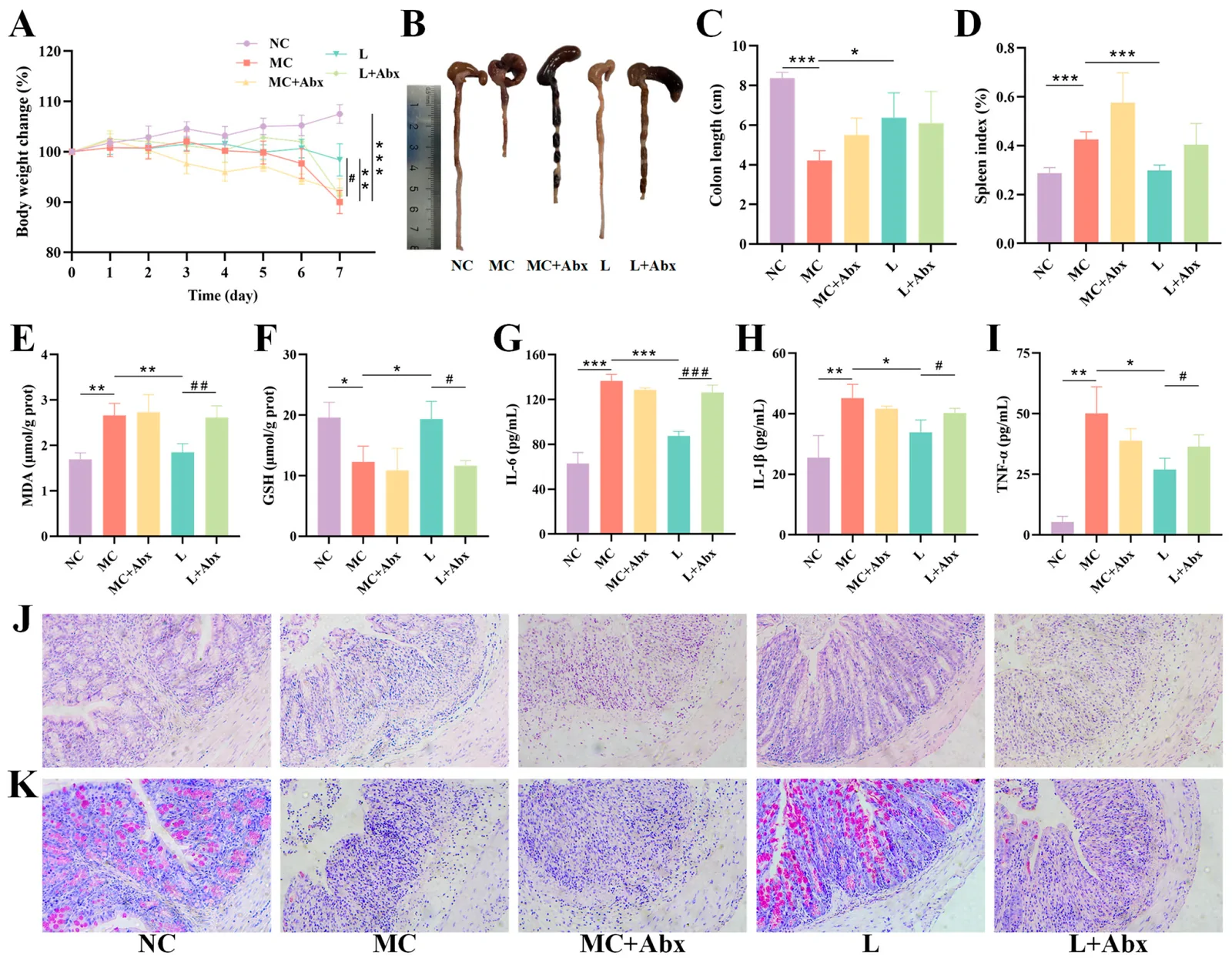
Gut microbiota depletion abolishes the therapeutic effects of LBTE against DSS-induced UC. (**A**) Body weight changes during DSS induction in mice with or without antibiotic treatment. (**B**,**C**) Representative images and statistical quantification of colon length. (**D**) Spleen index. (**E**,**F**) Levels of oxidative stress markers: MDA and GSH. (**G**–**I**) Levels of pro-inflammatory cytokines: IL-6, IL-1β, and TNF-α. (**J**,**K**) Representative histopathological micrographs based on H&E and PAS staining of colon sections (200 × magnification). Data are presented as mean ± SD. * *p* < 0.05, ** *p* < 0.01, *** *p* < 0.001 vs. the MC group. ^#^ *p* < 0.05, ^##^ *p* < 0.01, ^###^ *p* < 0.001 vs. the L + Abx group.

To investigate whether gut microbiota mediates the therapeutic efficacy of LBTE, a pseudo-germ-free (PGF) mouse model was established using a cocktail of broad-spectrum antibiotics (Abx). Successful microbiota depletion was verified by 16S rRNA sequencing of cecal contents. In Abx-treated mice, the microbial community was predominantly composed of Proteobacteria, with the normally dominant Firmicutes and Bacteroidota virtually absent (Figure 8A). No significant differences in microbial richness, diversity, or composition were observed between the MC + Abx and L + Abx groups (Figure 8B–E). Multivariate analyses (PCA, PCoA, and NMDS) demonstrated substantial overlap between the MC + Abx and L + Abx groups, confirming near-complete and uniform gut microbiota depletion (Figure 8F–H). In conventionally colonized (non-Abx) mice, low-dose LBTE significantly ameliorated DSS-induced colitis compared with the MC group, as evidenced by attenuated weight loss, preserved colon length, and reduced spleen index (Figure 9A–D; *p* < 0.05). Notably, the therapeutic effect of LBTE was largely abolished in the L + Abx groups. Accordingly, the L + Abx and MC + Abx groups showed no significant differences in oxidative stress markers (MDA and GSH) or pro-inflammatory cytokines (IL-6, TNF-α, and IL-1β) (Figure 9E–I). Histopathological examination further confirmed persistent epithelial erosion and goblet cell loss in both Abx-treated groups, demonstrating that an intact microbiota is essential for the mucosal protective effects of LBTE (Figure 9J,K). Collectively, these findings establish that the therapeutic efficacy of LBTE against UC is strictly contingent upon a functional gut microbiota.

### 3.10. FMT Recapitulates LBTE-Mediated Protection via Microbial and SCFA Homeostasis

**Figure 10:**
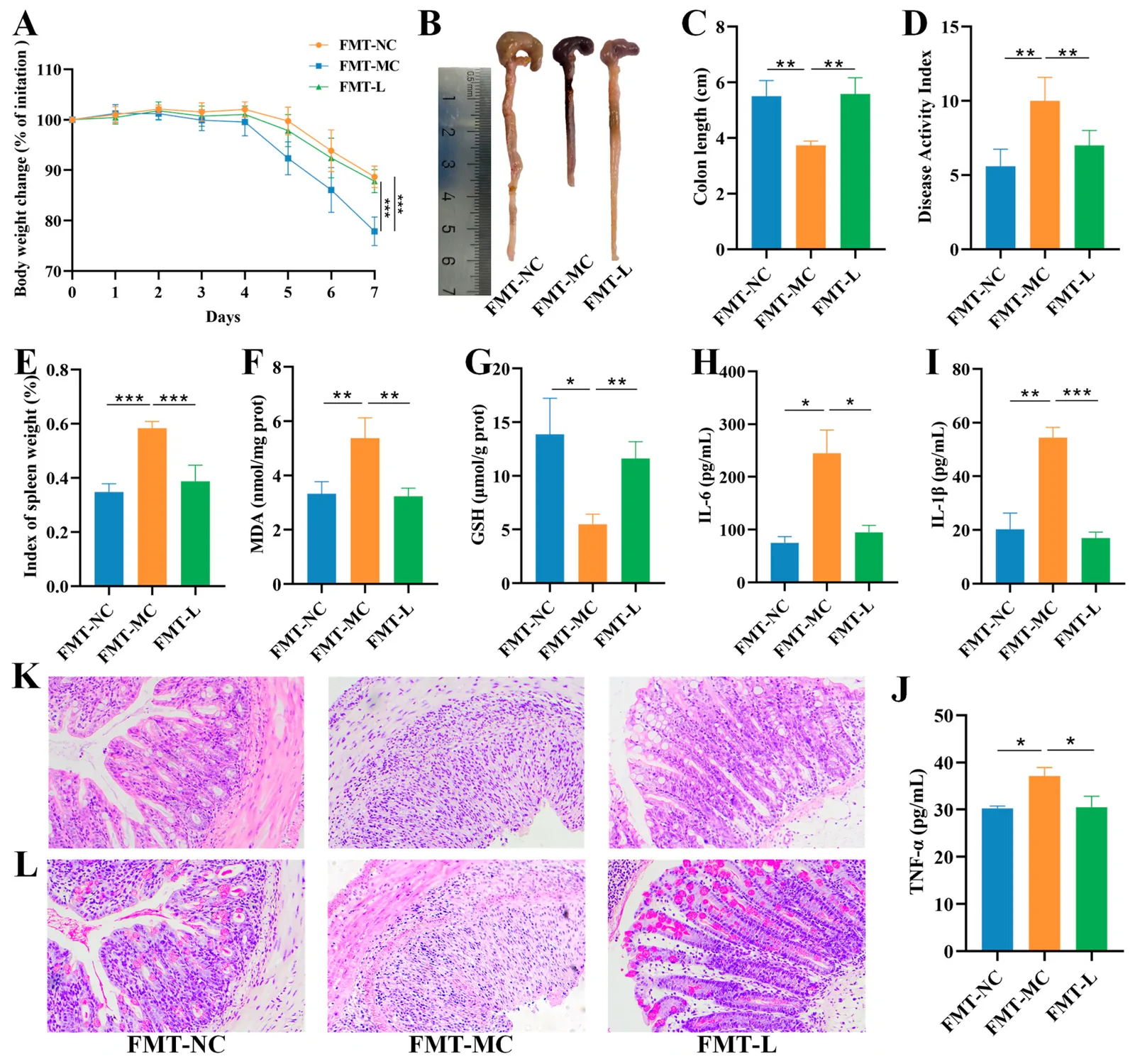
FMT from LBTE-treated donor recapitulates colonic protection in recipient mice. (**A**–**E**) Amelioration of clinical symptoms, including weight loss, colon shortening, DAI scores, and spleen index. (**F**,**G**) Levels of oxidative stress markers: MDA and GSH. (**H**–**J**) Levels of pro-inflammatory cytokines of IL-6, IL-1β, and TNF-α. (**K**,**L**) Histopathological confirmation of mucosal recovery (H&E and PAS staining). Data are expressed as mean ± SD. * *p* < 0.05, ** *p* < 0.01, *** *p* < 0.001 vs. the FMT-MC group.

To definitively validate whether the gut microbiota serves as a functional mediator of LBTE’s therapeutic effects, we performed a FMT experiment. Recipients of microbiota from LBTE-treated donors (FMT-L group) exhibited a marked attenuation of DSS-induced colitis symptoms compared to those receiving microbiota from untreated colitic donors (FMT-MC group). Specifically, compared with the FMT-MC group, the FMT-L group exhibited significantly attenuated weight loss, preserved colon length, and decreased spleen index and DAI scores (Figure 10A–E; *p* < 0.05), confirming the successful transfer of LBTE-mediated protection. Biochemical analyses further revealed that the FMT-L intervention markedly restored systemic redox balance and suppressed colonic inflammation, as evidenced by substantial decrease in MDA and pro-inflammatory cytokines (IL-1β, IL-6, and TNF-α) alongside a robust elevation in GSH levels (Figure 10F–J; *p* < 0.05). Histopathological examination confirmed that the FMT-L group maintained superior mucosal architecture and goblet cell density, whereas the FMT-MC group suffered from extensive epithelial erosion and massive inflammatory infiltration (Figure 10K,L). These findings provide compelling evidence that LBTE-conferred therapeutic effects can be horizontally transmitted via microbiota, reinforcing the causal relationship between microbial remodeling and disease management.

**Figure 11:**
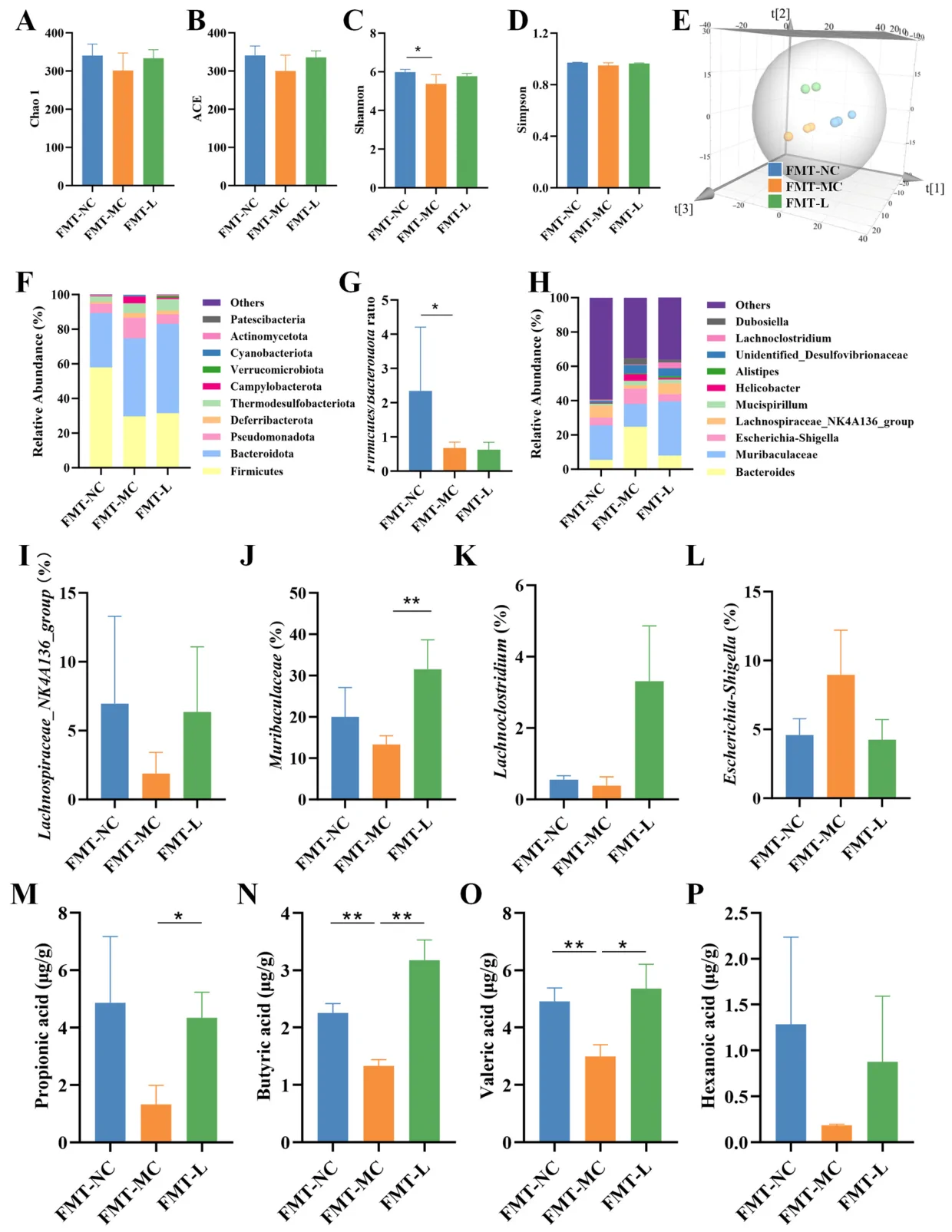
FMT from LBTE-treated donor mice promoted the production of gut microbiota-derived SCFAs. (**A**–**D**) α-diversity indices of the recipient gut microbiota, including Chao 1, ACE, Shannon, and Simpson. (**E**) OPLS-DA-based β-diversity at the OTU level. (**F**) Relative abundance of gut microbiota at phylum level. (**G**) *Firmicutes/Bacteroidota* ratio. (**H**) Relative abundance of gut microbiota at genus level. (**I**–**L**) Abundance of beneficial SCFA-producing genera: *Lachnospiraceae_NK4A136_group*, *Muribaculaceae*, *Lachnoclostridium*, and *Escherichia-Shigella*. (**M**–**P**) Quantitative analysis of cecal SCFA concentrations in recipient mice, including propionic acid, butyric acid, valeric acid, and hexanoic acid. Data are expressed as mean ± SD. * *p* < 0.05, ** *p* < 0.01 vs. the FMT-MC group.

16S rRNA sequencing of the recipient cecal contents further elucidated the underlying microbial shifts. Consistent with the above observations, α-diversity indices (Chao 1, ACE, and Shannon) indicated that FMT from LBTE-treated donors rescued the microbial richness and diversity compromised by DSS-induced UC mice (Figure 11A–D). OPLS-DA-based β-Diversity analysis revealed distinct clustering of microbial communities among various experimental groups (Figure 11E). At the phylum level, compared with the FMT-MC group, the FMT-L treatment group showed a trend toward increased relative abundance of *Firmicutes* and an elevated *Firmicutes*/*Bacteroidota* (F/B) ratio (Figure 11F,G). Notably, compared with the FMT-MC group, the FMT-L group exhibited not only enrichment of beneficial SCFA-producing bacteria, including *Lachnospiraceae_NK4A136_group*, *Muribaculaceae*, and *Lachnoclostridium*, but also reduction in the abundance of the pathogenic bacterium *Escherichia-Shigella* (Figure 11H–L). These genera are closely associated with epithelial barrier protection and mucosal immune regulation. Correspondingly, post-FMT metabolic analysis revealed a significant recovery in the concentrations of propionic acid, butyric acid, and valeric acid (Figure 11M–P; *p* < 0.05). The concomitant restoration of these metabolic byproducts of gut microbiota suggests that the anti-inflammatory effects of the transplanted microbiota are mainly driven by the re-establishment of SCFA metabolic homeostasis. Therefore, these results confirm that the gut microbiota and its associated SCFA metabolism are indispensable for mediating the therapeutic potential of LBTE against UC.

## 4. Conclusions

**Figure 12:**
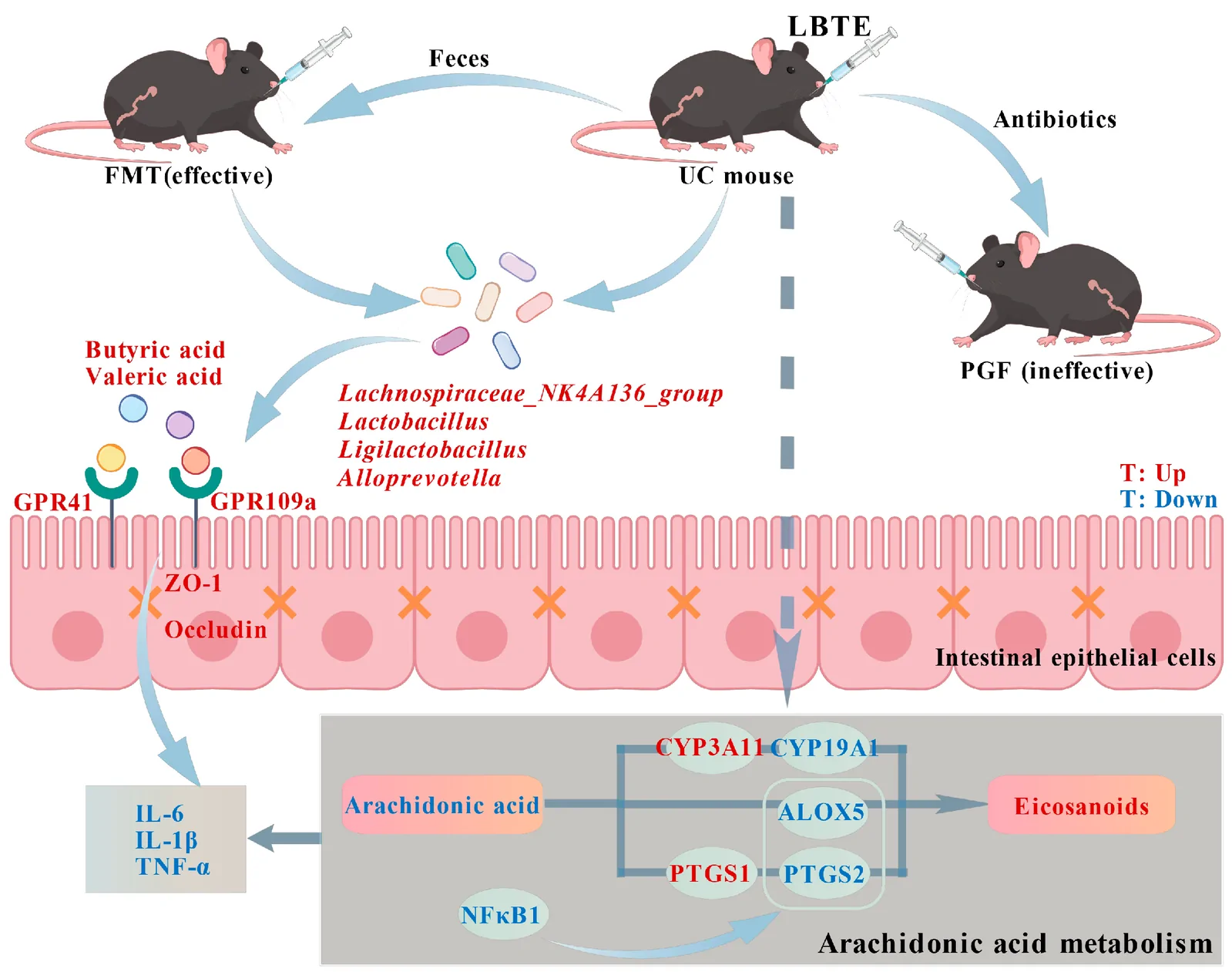
Schematic illustration of the integrated mechanisms by which LBTE alleviates UC.

In summary, this research systematically elucidated the therapeutic efficacy of LBTE against UC and deciphered its multifaceted underlying mechanisms (Figure 12). LBTE intervention robustly suppressed colonic inflammation and sealed mucosal injury, thereby facilitating the mechanical restoration of intestinal barrier integrity to decisively mitigate UC pathogenesis. Mechanistically, LBTE remodeled the gut microbial ecosystem by enriching beneficial SCFA-producing taxa, prominently including *Lachnospiraceae_NK4A136_group*, *Lactobacillus*, *Ligilactobacillus*, and *Alloprevotella*. This microbial shift directly led to a targeted upsurge in colonic butyric and valeric acid levels, which consequently reinforced the barrier function and preserved mucosal immune homeostasis. Furthermore, LBTE orchestrated a coordinated rebalancing of host arachidonic acid metabolism by modulating the expression of core genes, specifically *NFκB1*, *PTGS1/2*, *ALOX5*, *CYP3A11*, and *CYP19A1*, thereby suppressing the bio-synthesis of pro-inflammatory downstream eicosanoid signaling. Crucially, the complete abrogation of this therapeutic efficacy in PGF mice, coupled with the successful adoptive transfer of the protective phenotype via FMT, unequivocally underscores the indispensable and obligatory role of the commensal microbiota in driving LBTE-mediated colonic remediation. Collectively, LBTE exerts multi-component, multi-target, and microbiota-dependent protection against UC by synergistically modulating host arachidonic acid metabolism and microbiota-derived SCFA metabolism to restore colonic immunity and barrier homeostasis. This study provides novel mechanistic insights into LBTE against UC and underscores its application potential as a promising natural intervention for the prevention and management of UC.

## Data Availability

The original contributions presented in this study are included in the article/Supplementary Materials. Further inquiries can be directed to the corresponding authors.

## Supplementary Materials

The following supporting information can be downloaded at: https://www.mdpi.com/article/10.3390/foods15173085/s1, Figure S1: LC-MS-based profiles of serum pharmacochemistry analysis; Figure S2: Biological safety assessments of LBTE in mice; Figure S3: Multivariate statistical analysis of serum untargeted metabolomics; Figure S4: Heatmap of shared differential metabolites identified between NC vs. MC groups and MC vs. L groups; Figure S5: Correlation analysis between UC-related clinical factors and gut microbiota at genus level; Figure S6: Correlation analysis between differential metabolites (NC vs. MC groups) and gut microbiota at genus level; Figure S7: Correlation analysis between differential metabolites (L vs. MC groups) and gut microbiota at genus level; Table S1: Chromatographic conditions for serum pharmacochemistry and untargeted metabolomic analyses; Table S2: Mass spectrometry conditions for serum pharmacochemistry and untargeted metabolomic analyses; Table S3: Chromatographic conditions for SCFA quantification; Table S4: Mass spectrometric conditions for SCFA quantification; Table S5: Primer sequences for the RT-qPCR analysis; Table S6: The UHPLC-Q-TOF-MS/MS characterization of absorbed phytochemical components of LBTE identified within the post-administration serum profiles.
