# Supplementary material for: Microbiota-Dependent Alleviation of Ulcerative Colitis by Liubao Tea: Integrated Insights into SCFA and Arachidonic Acid Metabolism
Source: Foods. 2026 Aug 31;15(17):3085. doi: 10.3390/foods15173085 (PMC13565255; doi:10.3390/foods15173085)
Supplement: Supplementary file 1 [file foods-15-03085-s001.zip › foods-4473261-supplementary.pdf]

Supplementary Material

Microbiota-Dependent Alleviation of Ulcerative Colitis by Liubao Tea: Integrated Insights into SCFA and Arachidonic Acid Metabolism

Xiao Yang[1,3,†], Song Xu[2,†], Ying Tong[2], Jichu Luo[2], Xixing Fang[1,3], Jiaxing Du[1,3], Changyuan Zhou[1], Guangnian Hu[1,3], Bao Yang[1,3,*], Qisong Zhang[1,2,*]

[1] *Hubei Provincial Key Laboratory of Occurrence and Intervention of Rheumatic Diseases, Hubei Minzu University, Enshi, 445000, China.; yangx5679@163.com (X.Y); fangxixing2020@163.com (X.F.); dujiaxing1126@163.com (J.D.); 1067577144@qq.com (C.Z.); h997445219@163.com (G.H.); ybsept@hbmzu.edu.cn (B.Y.); zhangqisong@gxu.edu.cn (Q.Z.)*

[2] *Guangxi Key Laboratory of Special Biomedicine, School of Medicine, Guangxi University, Nanning, 530004, China; fighting68_xusong@163.com (S.X.); tongying0206@163.com (Y.T.); luojichu1999@163.com (J.L.); zhangqisong@gxu.edu.cn (Q.Z.)*

[3] *Medical School, Hubei Minzu University, Enshi, 445000, China*

* Correspondence: ybsept@hbmzu.edu.cn (B.Y.); Tel.: +86-0718-8206503; zhangqisong@gxu.edu.cn (Q.Z.); Tel.: +86-0771-3387105

† These authors contributed to the work equally

Figure. S1. LC-MS-based profiles of serum pharmacochemistry analysis.

Figure. S2. Biological safety assessments of LBTE in mice.

Figure. S3. Multivariate statistical analysis of serum untargeted metabolomics.

Figure. S4. Heatmap of shared differential metabolites identified between NC vs. MC groups and MC vs. L groups.

Figure. S5. Correlation analysis between UC-related clinical factors and gut microbiota at genus level.

Figure. S6. Correlation analysis between differential metabolites (NC vs. MC groups) and gut microbiota at genus level.

Figure. S7. Correlation analysis between differential metabolites (L *vs.* MC groups) and gut microbiota at genus level.

Table. S1. Chromatographic conditions for serum pharmacochemistry and untargeted metabolomic analyses.

Table. S2. Mass spectrometry conditions for serum pharmacochemistry and untargeted metabolomic analyses.

Table. S3. Chromatographic conditions for SCFA quantification.

Table. S4. Mass spectrometric conditions for SCFA quantification.

Table. S5. Primer sequences for the RT-qPCR analysis.

Table. S6. The UHPLC-Q-TOF-MS/MS characterization of absorbed phytochemical components of LBTE identified within the post-administration serum profiles.

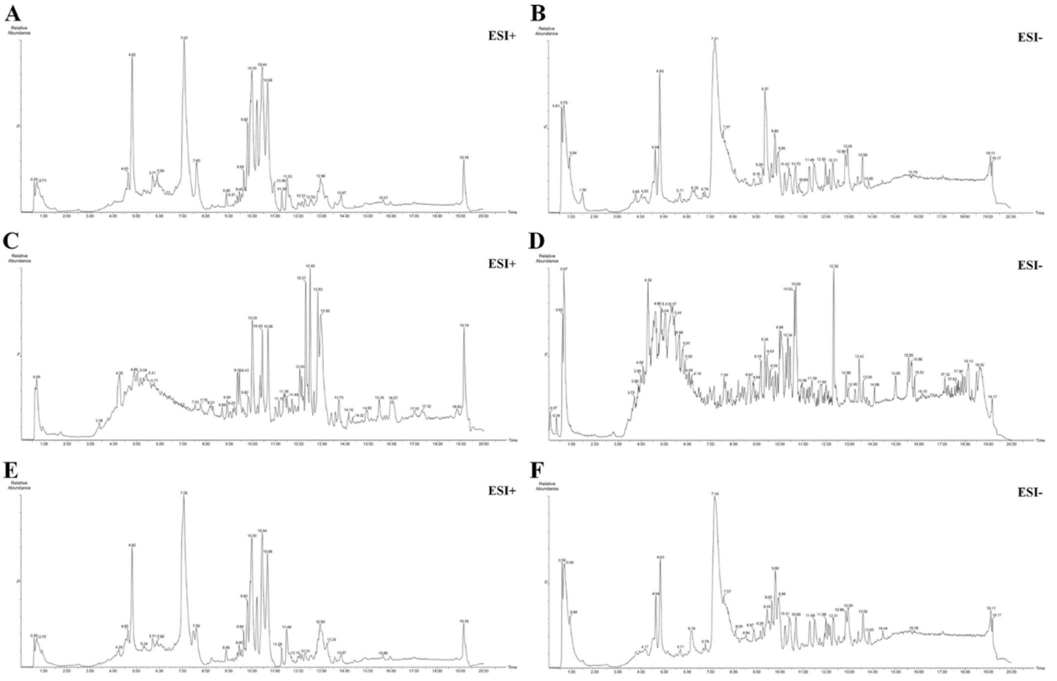

Figure. S1. LC-MS-based profiles of serum pharmacochemistry analysis. (A, B) Total ion chromatograms of blank serum in ESI+ and ESI- modes. (C, D) Total ion chromatograms of LBTE in ESI+ and ESI- modes. (E, F) Total ion chromatograms of serum containing LBTE in ESI+ and ESI- modes.

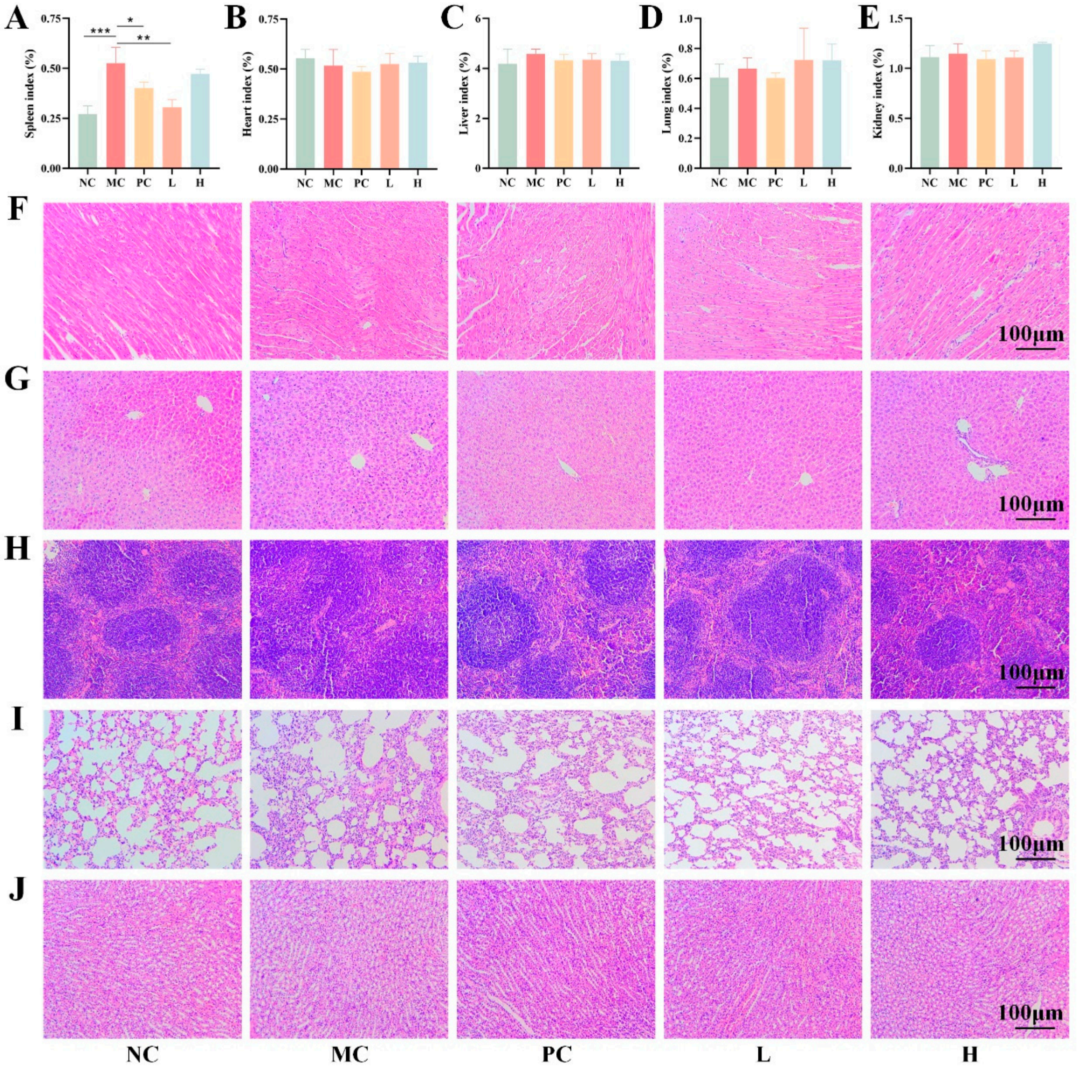

Figure. S2. Biological safety assessments of LBTE in mice. (A–E) Main organ indexes. Representative H&E-stained sections of (F) heart, (G) liver, (H) spleen, (I) lung, and (J) kidney sections (200 × magnification).

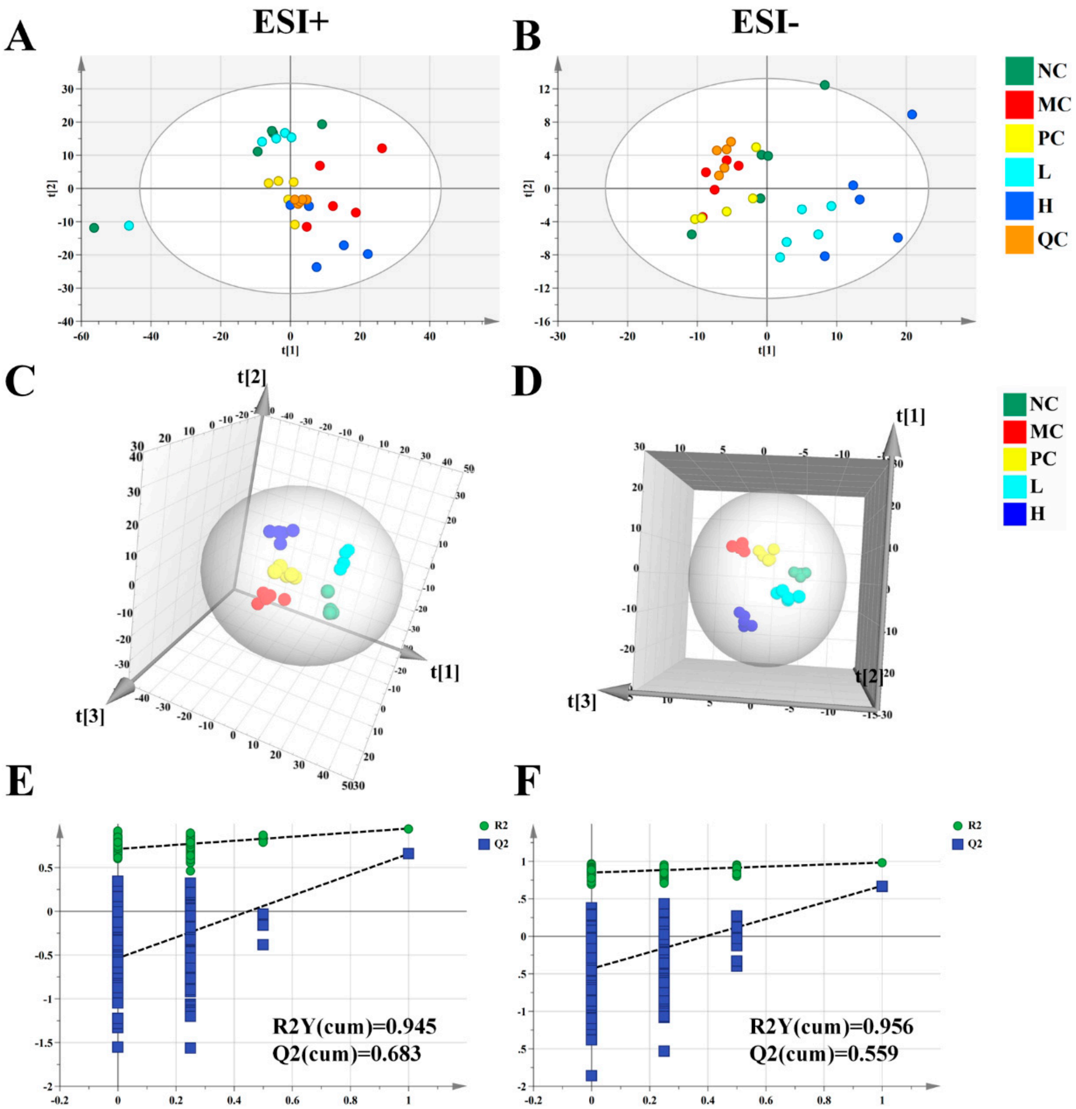

Figure. S3. Multivariate statistical analysis of serum untargeted metabolomics. (A, B) PLS-DA score plots in ESI+ and ESI- modes; (C, D) OPLS-DA score plots in ESI+ and ESI- modes. (E, F) Permutation test validation (200 times) of the OPLS-DA model in ESI+ and ESI- modes.

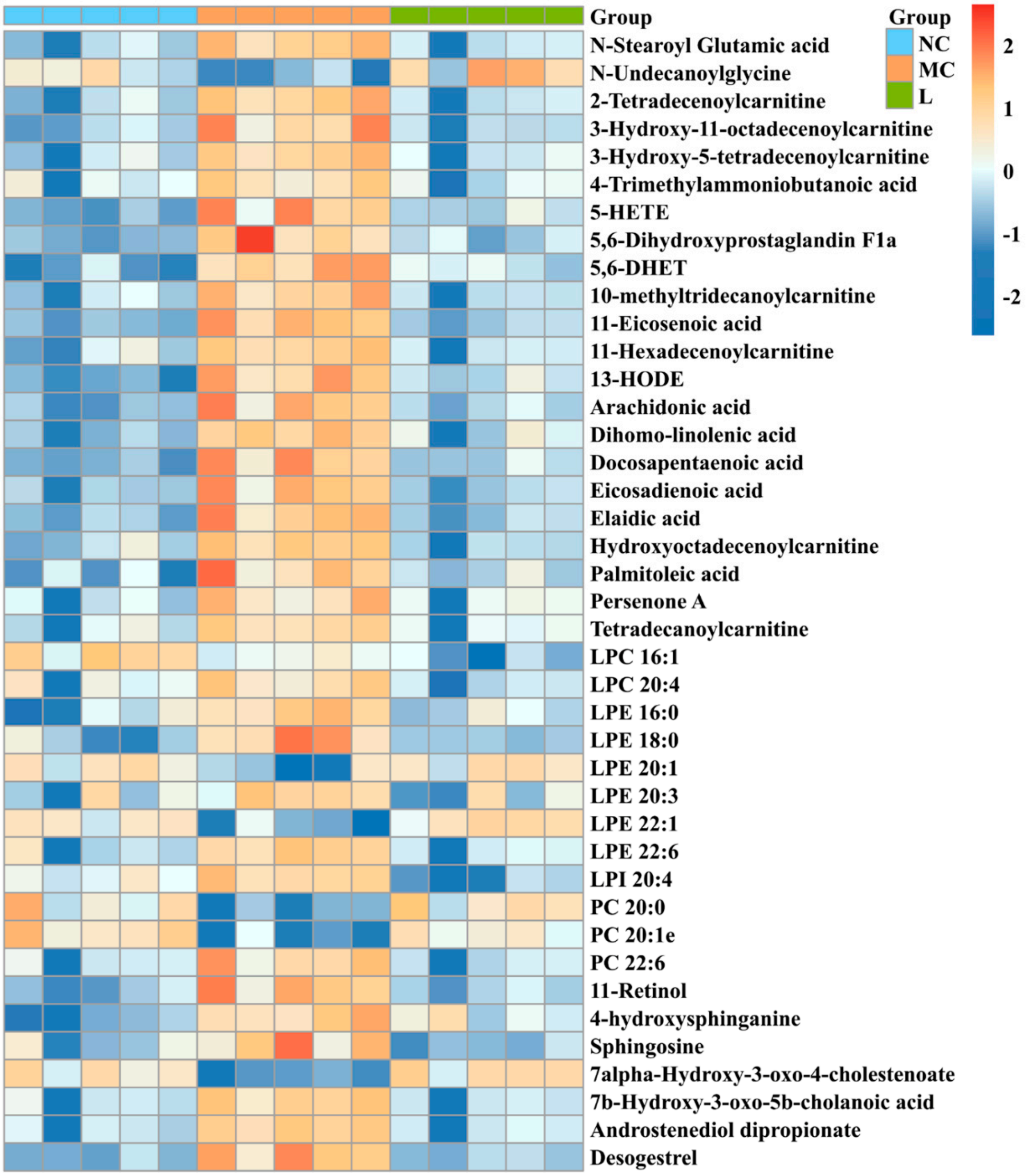

Figure. S4. Heatmap of shared differential metabolites identified between NC *vs.* MC groups and MC *vs.* L groups.

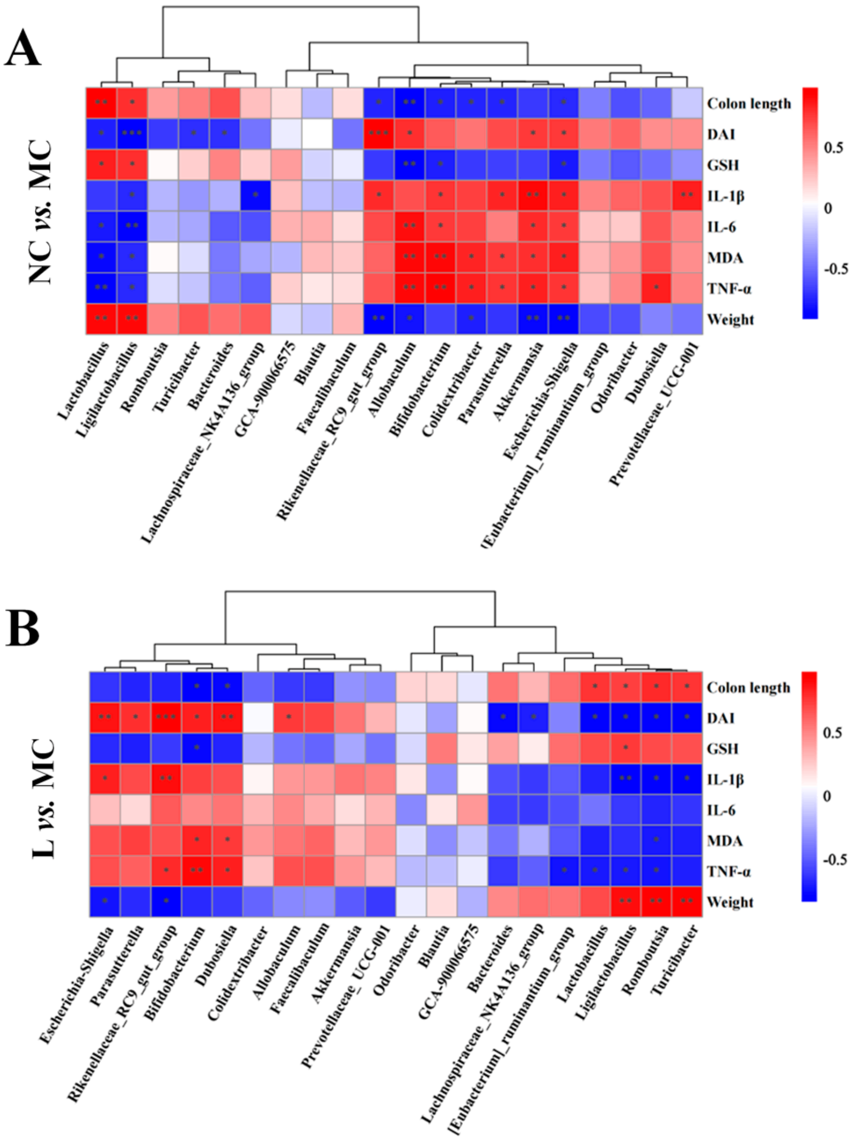

Figure. S5. Correlation analysis between UC-related clinical factors and gut microbiota at genus level.

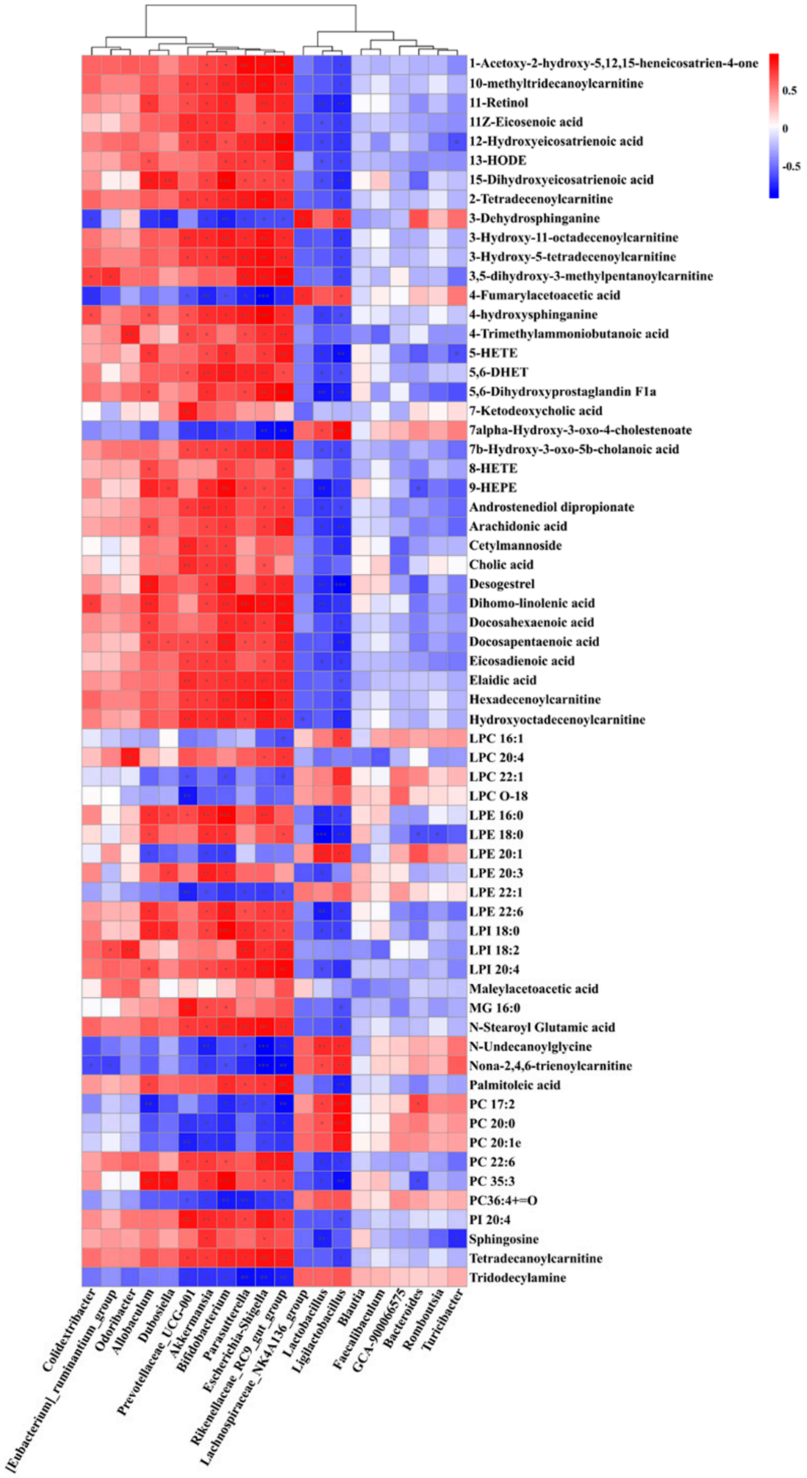

Figure. S6. Correlation analysis between differential metabolites (NC *vs.* MC groups) and gut microbiota at genus level.

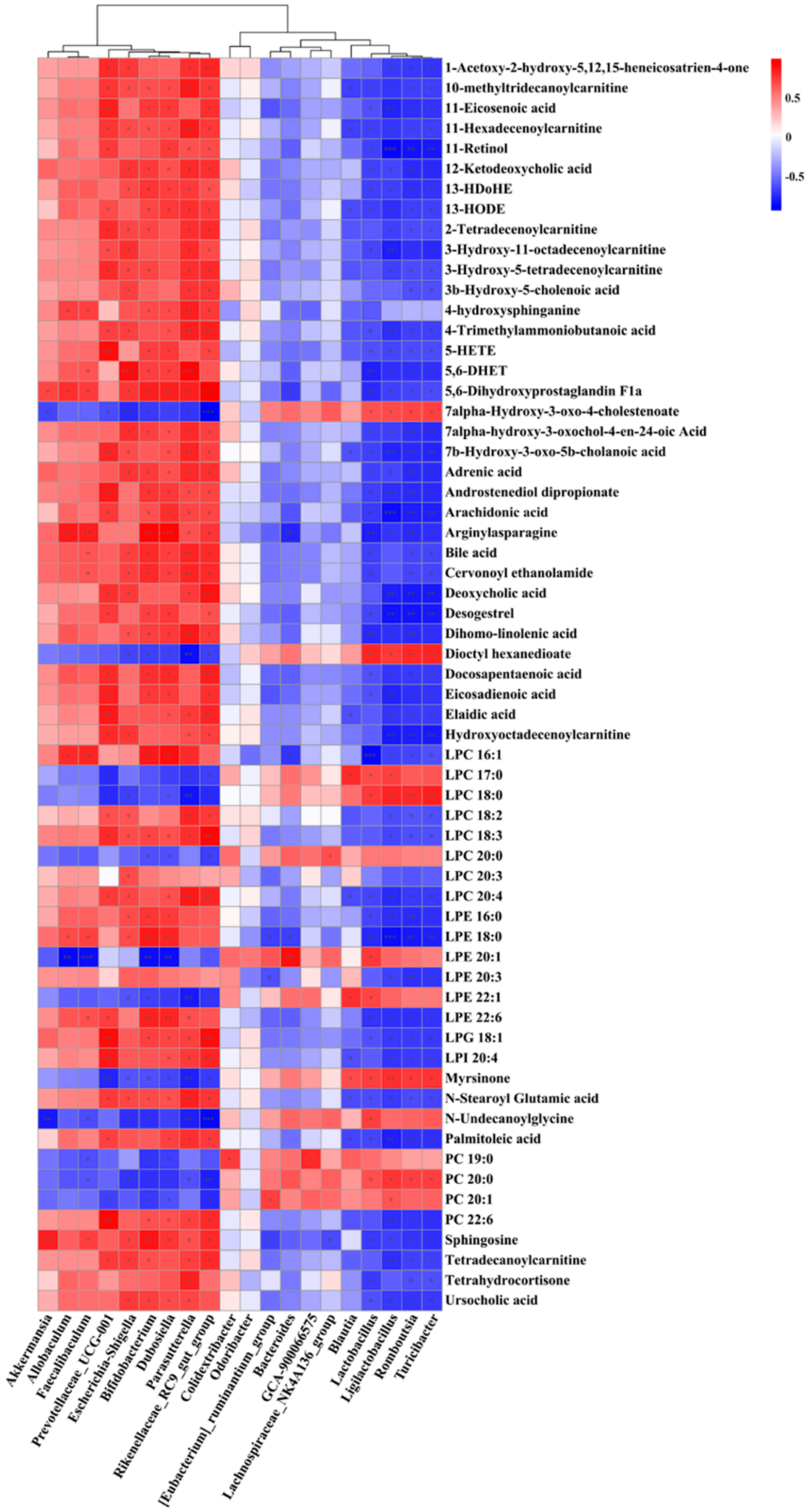

Figure. S7. Correlation analysis between differential metabolites (L *vs.* MC groups) and gut microbiota at genus level.

Table. S1. Chromatographic conditions for serum pharmacochemistry and untargeted metabolomic analyses.

Time (min)	Flow rate (mL/min)	Mobile phase A (%)	Mobile phase B (%)	Injection volume (μL)
0	0.4	98	2	
2	0.4	98	2	
13	0.4	0	100	5
18	0.4	0	100	
20	0.4	98	2	

Analytical conditions: Waters Acquity UPLC BEH C18 (1.7 μm, 2.1 mm × 150 mm) at 40°C column temperature. Mobile phases: A was ultrapure water and B was acetonitrile, both of which contained 0.01% formic acid in ESI+ and ESI- modes.

Table. S2. Mass spectrometry conditions for serum pharmacochemistry and untargeted metabolomic analyses.

Parameters	Values
Capillary voltage (V)	2000
Cone (V)	40
Source temperature (°C)	150
Desolvation temperature (°C)	500
Cone gas (L/h)	50
Desolvation gas (L/h)	1000
Nebuliser gas (bar)	6
Scan mode	Full scan+MS2
Scan range (*m/z*)	100-1200

Table. S3. Chromatographic conditions for SCFA quantification.

Time (min)	Flow rate (mL/min)	Mobile phase A (%)	Mobile phase B (%)	Injection volume (μL)
0	0.2	90	10	
15	0.2	5	95	
27	0.2	5	95	10
35	0.2	90	10	
40	0.2	90	10	

Analytical conditions: Waters Acquity UPLC HSS T3 (1.8 μm, 2.1 mm × 100 mm) at 40°C column temperature. Mobile phases: A was ultrapure water and B was acetonitrile, both of which contained 0.01% formic acid in ESI+ mode.

Table. S4. Mass spectrometric conditions for SCFA quantification.

Parameters	Values
Capillary voltage (V)	3500
Fragmentor voltage (V)	50
Sheath gas flow (L/min)	11
Gas flow (L/min)	8
Gas temperature (°C)	320
Sheath gas temperature (°C)	350
Nebulizer pressure (psi)	50
Scan mode	Full scan
Scan range (*m/z*)	100-1700

Table. S5. Primer sequences for the RT-qPCR analysis.

Gene	Forward Primer (5′-3′)	Reverse Primer (5′-3′)
GAPDH	AGGTCGGTGTGAACGGATTTG	TGTAGACCATGTAGTTGAGGTCA
NFκB1	GAACAAGAAATCCTACCCAC	ATACCCCGTCCTCACAGT
ALOX5	TACCTCAGCCTCATTGGCTCT	TGGAGCCAGTATTTGCGCTTC
PTGS1	GATTGGTGGAGGTAGGAAC	CATTGAAGGGCTGTAGGC
PTGS2	TTGCATTCTTTGCCCAGCACT	ACCTCTCCACCAATGACCTGAT
CYP3A11	CAGAAGGCAAAGAAAGGC	GTTGAGGGAATCCACGTT
CYP19A1	CATACTGGAGATGCTGATTG	CTGGATACTCTGCGACGA
ZO-1	GCGAACAGAAGGAGCGAGAAGAG	GCTTTGCGGGCTGACTGGAG
Occludin	TGGCTATGGAGGCGGCTATGG	AAGGAAGCGATGAAGCAGAAGGC
GPR41	TTCCTCCAAGTTCCAAGCCG	GGCTGACTTGCTGAGTCCAA
GPR109a	TCCAAGTCTCCAAAGGTGGT	TGTTTCTCTCCAGCACTGAGTT

Table. S6. The UHPLC-Q-TOF-MS/MS characterization of absorbed phytochemical components of LBTE identified within the post-administration serum profiles.

NO.	Compounds	RT (min)	Adduct	Observed mass	ppm	Formula	Fragment ions
1	Suberenone	0.94	M+Na	267.0616	4.98	$C_{14}H_{12}O_4$	154.9706, 203.0547
2	Apo-13-zeaxanthinone	10.57	M+K	313.1577	4.58	$C_{18}H_{26}O_2$	265.2552, 299.1408
3	Tiptolide	3.08	M+H	361.1652	1.87	$C_{20}H_{24}O_6$	251.0566, 253.1453
4	Romucosine H	4.26	M+Na	408.1456	9.86	$C_{21}H_{23}NO_6$	138.0677, 195.0897
5	Crysoeriol 6-hexoside	4.26	M+Na	473.1241	7.62	$C_{22}H_{26}O_9$	195.0897, 457.1469
6	6-Hydroxypentadecanedioic acid	5.20	M+Na	311.1854	8.85	$C_{15}H_{28}O_5$	291.0995, 298.2036
7	Epirosmanol	9.58	M+H	347.1864	3.06	$C_{20}H_{26}O_5$	304.3027, 313.2398
8	Secoisolariciresinol	8.30	M-H	361.1671	3.93	$C_{20}H_{26}O_6$	221.1568, 328.1680
9	Isofucosterol glucoside	9.80	M+FA-H	619.4167	8.37	$C_{35}H_{58}O_6$	279.2359, 395.2439, 504.3092
10	Epigallocatechin	4.95	M+FA-H	351.0733	3.79	$C_{15}H_{14}O_7$	283.0853, 341.0445
11	Trilobinol	11.71	M+FA-H	345.2085	4.51	$C_{20}H_{28}O_2$	301.2173, 339.2033
